# CRISPR-Cas13d mediates robust RNA virus interference in plants

**DOI:** 10.1186/s13059-019-1881-2

**Published:** 2019-12-02

**Authors:** Ahmed Mahas, Rashid Aman, Magdy Mahfouz

**Affiliations:** Laboratory for Genome Engineering and Synthetic Biology, Division of Biological Sciences, 4700 King Abdullah University of Science and Technology, Thuwal, 23955-6900 Saudi Arabia

**Keywords:** CRISPR-Cas, Cas13, RNA interference, Virus interference, Virus resistance, CasRx

## Abstract

**Background:**

CRISPR-Cas systems endow bacterial and archaeal species with adaptive immunity mechanisms to fend off invading phages and foreign genetic elements. CRISPR-Cas9 has been harnessed to confer virus interference against DNA viruses in eukaryotes, including plants. In addition, CRISPR-Cas13 systems have been used to target RNA viruses and the transcriptome in mammalian and plant cells. Recently, CRISPR-Cas13a has been shown to confer modest interference against RNA viruses. Here, we characterized a set of different Cas13 variants to identify those with the most efficient, robust, and specific interference activities against RNA viruses *in planta* using *Nicotiana benthamiana*.

**Results:**

Our data show that LwaCas13a, PspCas13b, and CasRx variants mediate high interference activities against RNA viruses in transient assays. Moreover, CasRx mediated robust interference in both transient and stable overexpression assays when compared to the other variants tested. CasRx targets either one virus alone or two RNA viruses simultaneously, with robust interference efficiencies. In addition, CasRx exhibits strong specificity against the target virus and does not exhibit collateral activity *in planta*.

**Conclusions:**

Our data establish CasRx as the most robust Cas13 variant for RNA virus interference applications *in planta* and demonstrate its suitability for studying key questions relating to virus biology.

## Introduction

Plant viruses are obligate parasites that rely mostly on host cells to complete their life (infection) cycle. They infect many economically important crops, leading to a significant decrease in crop yields worldwide. It is estimated that plant diseases cause 10 to 15% reduction in global crop yields each year, and 47% of this loss is caused by viruses [1–3]. Therefore, plant viruses threaten world agriculture and the food security of the rapidly growing world population. Most viruses infecting plants are RNA viruses, which comprise diverse groups and subgroups that are classified based on phylogenetic relationships determined by sequence homologies among the conserved virus genes, such as RNA-dependent RNA polymerase (RdRp), coat protein (CP), and movement protein (MP) [4]. Viruses with positive-sense, single-stranded RNA genomes represent most of these viruses. Therefore, we urgently need to develop effective antiviral agents to preserve crop yields from such viruses [5]. Various strategies, based on genome and genetic engineering approaches, have been developed to combat viruses, including directly inhibiting virus replication or targeting host factors required by viruses to replicate and persist [6]. RNA interference (RNAi), for example, is an innate antiviral immunity mechanism that has been successfully used to combat various plant viruses [7, 8]. Nevertheless, the availability of such antiviral strategies is still limited to specific virus groups, and many viruses, through evolution, mutate readily and have developed various counter-defense mechanisms, leading to rapid emergence of new viruses and nullifying the available antiviral approaches.

Bacterial and archaeal species employ CRISPR-Cas systems to fend off foreign genetic elements from invading phages and nucleic acids [9]. CRISPR-Cas adaptive immunity involves three main stages: (1) adaptation and spacer acquisition, where a piece of the invader genome is incorporated into the CRISPR array proximal to the 5′ leader sequence; (2) biogenesis of the CRISPR array into pre-CRISPR RNA (crRNA) to provide targeting specificity and processing into mature crRNA; and (3) interference, where the crRNA binds and guides the effector protein to the invader’s genome for cleavage or degradation [10]. These systems are divided into two classes: class I systems, which rely on multiple Cas proteins that mediate the target interference, and class II systems, which are represented by single, multidomain effector proteins. The class II CRISPR/Cas systems include types II, V, and VI. Types II and V comprise endonucleases that operate at the DNA level. On the other hand, type VI CRISPR/Cas systems are distinct in that they exclusively target RNA molecules [10, 11].

Because class II CRISPR-Cas systems are composed of a single effector to mediate the interference and immunity against the invader’s genomes, their engineering is simple; therefore, they are widely adopted in genome and transcriptome engineering. In recent years, class II CRISPR/Cas systems have been extensively developed to provide powerful and versatile tools for various genome engineering purposes [12–14]. Several studies have interrogated genomic and metagenomic sequencing data through the use of signature proteins to identify novel RNA-guided DNA and RNA endonucleases [11, 15]. Class II type II CRISPR-Cas9 targets DNA and has been used for genome engineering across eukaryotic species [16]. The impressive success of the DNA-targeting CRISPR/Cas systems in engineering and specifically editing the genome of different eukaryotic cells has led to the exploitation of this technology as a programmable antiviral defense strategy to confer resistance to many eukaryotic viruses, including human viruses [17–19]. In plants, CRISPR/Cas9 efficiently confers viral resistance to host plants. We and others have shown that CRISPR/Cas9 mediates strong interference against various DNA viruses. We have also shown that a single guide RNA targeting a conserved intergenic region in multiple Gemini viruses guides Cas9 to target different viruses simultaneously, providing interference against these DNA viruses [20–23]. These studies demonstrate the enormous potential of CRISPR/Cas9 as a promising strategy against plant DNA viruses [17]. Nevertheless, such DNA-targeting CRISPR modalities cannot target RNA molecules and therefore limit their applicability for targeted interference against RNA viruses.

Class II type VI CRISPR-Cas systems are RNA-guided and RNA-targeting machineries that provide prokaryotes with immunity against RNA [24] and DNA phages [25]. All type VI CRISPR-Cas systems have a single effector protein known as the Cas13 effector, formerly the C2c2 CRISPR-Cas system [24]. Cas13 effectors possess two distinct catalytic activities; one is RNase activity provided by the two higher eukaryotic and prokaryotic nucleotide binding domains (HEPN), which is required for target RNA degradation, and the other is RNase activity that catalyzes the processing and maturation of the pre-crRNA into mature crRNA [26, 27]. Intriguingly, these systems, once activated through the specific binding of the target RNA, confer collateral “promiscuous” activity and non-specifically degrade the RNA transcripts in the cell [24]. This collateral activity was harnessed to develop highly sensitive pathogen detection methods [28–31].

CRISPR/LshCas13a from *Leptotrichia shahii* was the first Cas13 orthologue to be harnessed for programmable RNA-targeting activities [24]. We previously developed *Lsh*Cas13a to engineer immunity against the tobacco turnip mosaic RNA virus (TuMV) in different plant species, including *Nicotiana benthamiana* and *Arabidopsis thaliana* [32, 33]. We demonstrated that LshCas13a mediates specific RNA virus targeting in plants, albeit with moderate efficiency. In addition, we demonstrated that LshCas13a can process pre-crRNA to generate mature and functional crRNAs that guide LshCas13a to degrade the targeted virus. Subsequently, LshCas13a has become the prominent Cas13 orthologue in engineering immunity against different RNA viruses in different plant species, including monocot and dicot plants [34, 35]. Since the first report of LshCas13a, several studies have identified more variants of Cas13 proteins belonging to different Cas13 families, which have been classified into four type VI subtypes (A–D) [36–38]. These other variants have shown more robust catalytic activity and specificity compared to LshCas13a and have been harnessed for different RNA targeting and manipulations [28, 36, 38–40]. In addition, these studies have shown that these Cas13 variants exhibit variable efficiencies for transcriptome targeting and engineering. LwaCas13a, for example, has been reported to mediate more robust RNA-targeting activity than the LshCas13 system but requires a stabilizer fusion, for example, msfGFP for efficient interference activity [38]. Subsequent studies have identified PspCas13b to be more efficient for RNA targeting in mammalian cells compared to the LwaCas13a protein, and PspCas13b does not require a stabilizer protein for its activity [39]. Recently, a new and additional Cas13 subtype has been identified, type VI-D, which shows minimal sequence identity with previous Cas13 effectors. Interestingly, Cas13d effector proteins are much smaller than all previously reported subtypes (median size ~ 300 amino acids, 26% smaller than other Cas13 proteins) [36, 40] and similar to type VI-B; the majority of the type VI-D orthologues contain associated accessory proteins harboring WYL domains [36]. Notably, experimental characterization of the Cas13d activity of the *Ruminococcus flavefaciens* (CasRx) orthologue has shown consistently robust activity and specificity in mammalian cells compared to the previously characterized Cas13 proteins, namely LwaCas13a and PspCas13b [40]. These enzymes will enable diverse RNA manipulations for different purposes.

Because most viruses infecting plants possess RNA genomes, and reasoning that other uncharacterized Cas13 proteins might exhibit more robust activity in plants against RNA viruses, we sought to identify a more robust RNA-targeting CRISPR/Cas13 system by characterizing a set of Cas13 family members to assess their virus RNA-targeting activity *in planta*. Here, we investigated nine different Cas13 variants for robust virus interference to establish the use of this adaptive immunity mechanism for engineering plant immunity against single and multiple RNA viruses. We identified CasRx as the most effective Cas13 orthologue for RNA virus targeting in *N. benthamiana*. CasRx confers robust RNA virus interference compared to other variants in transient assays targeting a highly replicating RNA virus. In addition, transgenic plants overexpressing CasRx exhibited efficient interference against TuMV RNA compared to plants overexpressing other Cas13 variants, including LwaCas13a and PspCas13b, using cognate, position-matched crRNAs. Importantly, the catalytically deactivated (dCasRx) version could not mediate any virus targeting and, thus, inhibition, indicating that the HEPN-dependent catalytic activity of CasRx is required for virus RNA degradation. Finally, using synchronous coinfection of two non-competing RNA viruses, we show that CasRx RNA virus targeting is highly specific to the targeted virus and lacks collateral activities *in planta*. In addition, CasRx mediated efficient multiplexed virus interference by targeting two different RNA viruses simultaneously. This work establishes the use of CasRx for efficient RNA virus interference applications in plants.

## Results

### Construction of different CRISPR-Cas13 variants for expression *in planta*

**Fig. 1:**
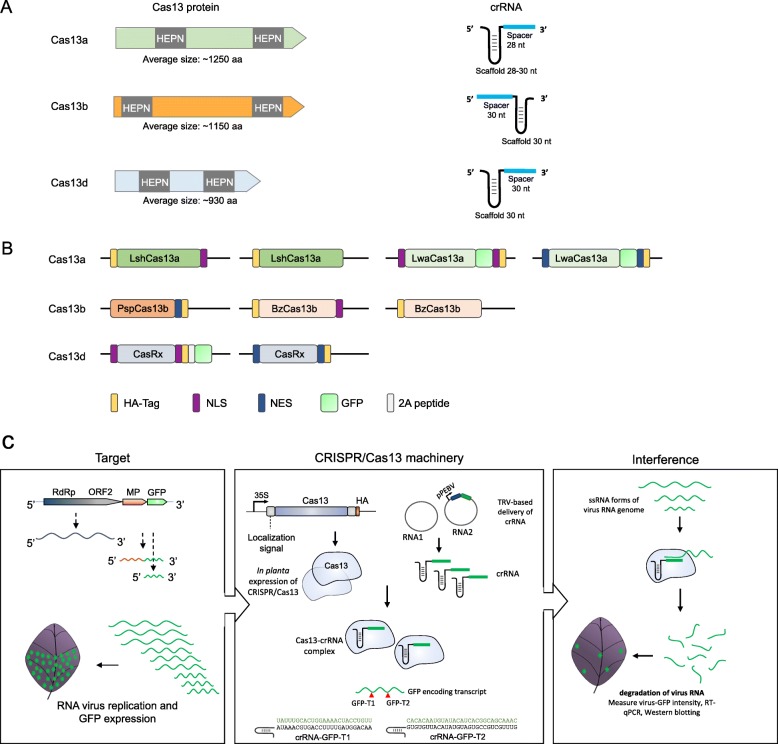
CRISPR/Cas13 orthologues for RNA virus interference. **a** Schematic representation of CRISPR/Cas13 variants and their respective crRNA structures. Structural representations of different Cas13 subtypes with their corresponding crRNA structures are shown with the estimated average size of the Cas13 protein under each subtype. HEPN, high eukaryotic and prokaryotic nucleotide binding domains; aa, amino acid; nt, nucleotides. **b** Schematic of different Cas13 protein variants used in this study. The different Cas13 variants are shown with their different fusions. NLS, nuclear localization signal; NES, nuclear export signal; GFP, green fluorescent protein. **c** Schematic of TRBO-based Cas13 mediated RNA virus interference. The highly replicating plant TRBO RNA virus expressing GFP protein was used as a reporter system to screen for efficient Cas13 activity in transient assays via crRNA delivered through TRV system, targeting two different regions within the GFP sequence of the virus RNA genome. 35S, CaMV 35S promoter; pPEBV, pea early browning virus promoter; RNA1/RNA2, genomes of the tobacco rattle virus (TRV)

Different CRISPR-Cas13 variants have been identified through computational pipelines searching for effector proteins possessing two HEPN domains. Cas13a, Cas13b, and Cas13d have been identified and tested for RNA manipulation in vitro and in vivo [36–38, 40–42]. Here, we investigated different Cas13 orthologues to assess their catalytic activities for RNA virus interference in plants. To this end, we generated binary vectors for *in planta* expression of multiple Cas13 proteins of three different Cas13 subtypes, including subtypes A, B, and D (Fig. 1a). In addition to the LshCas13a that we previously employed [32, 33], we selected one orthologue of Cas13a from *Leptotrichia wadei* (LwaCas13a), two orthologues of Cas13b from *Bergeyella zoohelcum* (BzCas13b) and from *Prevotella sp. P5-125 (*PspCas13b), and one orthologue of Cas13d from *Ruminococcus flavefaciens* XPD3002 (CasRx). The selection of these different Cas13 orthologues was based on several reports showing improved RNA-targeting activity of these variants in comparison to LshCas13a protein in vitro and in mammalian cells [37, 38, 40, 42]. Different fusions were made to these Cas13 variants to improve their stability or control their subcellular localization for optimum activity against the target transcripts. The different Cas13 protein open reading frames (ORFs) were fused to either a nuclear localization signal (NLS) or a nuclear export signal (NES), or not fused to any localization signal. In addition, all proteins were fused with an HA tag to facilitate protein detection (Fig. 1b). The different Cas13 orthologues were cloned into the *pK2GW7* binary vector, and their expression was driven by the 35S cauliflower mosaic virus promoter. Because the PspCas13b orthologue has been previously shown to be most active for RNA interference with the NES signal [39], we proceeded with the PspCas13b-NES module without considering an NLS variant. For expression of Cas13’s cognate crRNAs conferring specificity against the green fluorescent protein (GFP) reporter gene in the target virus or against a virus protein-coding sequence, we employed our tobacco rattle virus (TRV)-based system to transiently and systemically express the crRNAs in plants [43], where the corresponding crRNAs were cloned under the pea early browning virus promoter, pPEBV, in the TRV-RNA2 genome.

### Screening of Cas13 variants for efficient RNA virus interference in transient assays

To assess the CRISPR/Cas13 activity *in planta*, generation of transgenic plants overexpressing the CRISPR/Cas13 machinery is normally needed. However, such an approach is time-consuming and challenging when multiple and different CRISPR/Cas13 variants need to be evaluated, especially when considering the evaluation of multiple and different crRNAs for each variant. Therefore, the development of a transient assay that allows the assessment of different Cas13 variants and crRNAs simultaneously and systemically prior to the generation of transgenic plants would be valuable. We adopted a tobacco mosaic virus (TMV)-RNA-based overexpression (TRBO-G) system expressing the GFP gene to serve as a reporter construct to measure Cas13 interference activity in transient assays in plants. TRBO-G is a positive-sense, single-stranded RNA plant-infecting virus that has been engineered to replace the CP-encoding sequence with a GFP-encoding sequence, rendering the virus unable to move systemically in the infected plants, but retaining its ability to highly and efficiently replicate and express GFP protein in the infected (infiltrated) leaves (Additional file 1: Figure S1) [44]. This reporter system was employed to provide a unique and easy-to-monitor system for virus infection and replication, thereby facilitating the assessment and comparison of the interference activity of the different CRISPR/Cas13 systems against RNA viruses in plants (Fig. 1c).

**Fig. 2:**
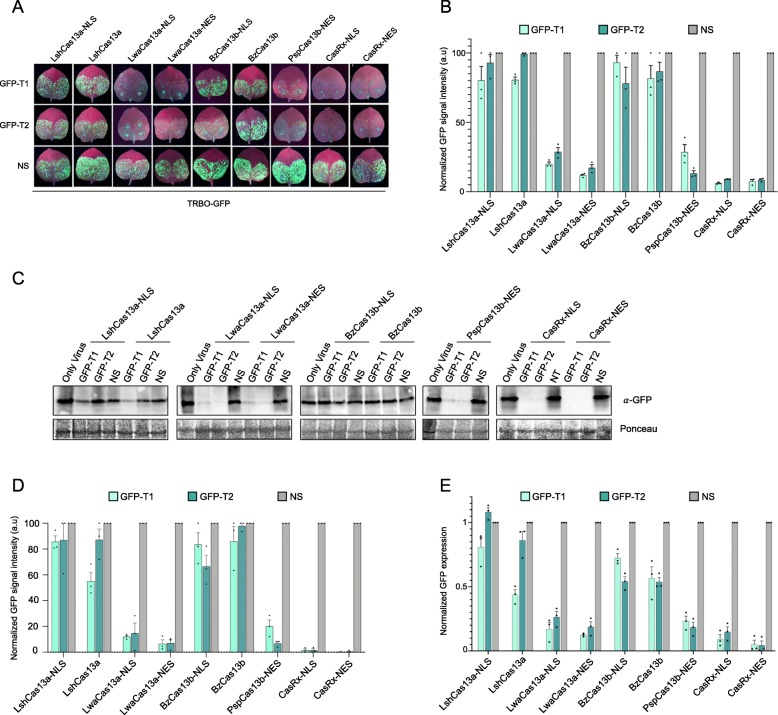
TRBO-GFP-based screening of different Cas13 orthologues for efficient virus interference. **a** GFP monitoring to assess the Cas13-mediated virus interference activities in *Agro*-infiltrated wild type *N. benthamiana* leaves in transient assays. Images were taken 3 days post-infiltration. NS, non-specific crRNA. **b** Relative fluorescence intensity quantification of leaf images. For each Cas13 variant, GFP signal intensity of each targeting crRNA (GFP-T1 and GFP-T2) is shown relative to the non-targeting (NS) crRNA. Values shown as mean ± SEM (*n* = 3). **c** Western blot analysis of the abundance of the virus expressed GFP protein to confirm the Cas13-mediated TRBO-GFP virus interference. Protein blots were developed with anti-GFP antibody. *α*-GFP, anti-GFP antibody. Ponceau staining served as loading control. **d** Quantification of the western blot data. For each Cas13 variant, the abundance of the GFP protein with the targeting crRNAs (GFP-T1 and GFP-T2) is shown relative to the non-targeting (NS) crRNA. Error bars indicate SEM (*n* = 3). **e** RT-qPCR analysis of TRBO-GFP knockdown with different Cas13 variants using the two position-matched crRNAs. For each Cas13 variant, knockdown efficiency of each targeting crRNA (GFP-T1 and GFP-T2) is shown relative to the non-targeting (NS) crRNA. Values shown as mean ± SEM (*n* = 3)

To assay for the interference activity of the different Cas13 variants, we co-delivered each of the Cas13 variant with the position-matched Cas13’s cognate crRNAs targeting GFP coding sequence within the TRBO-G genome, and the TRBO-G-expressing construct into leaves of wild type *N. benthamiana* plants via *Agro*-infiltration. We used a non-specific crRNA with no complementarity to the TRBO-G genome as a negative control. We tested two position-matched crRNAs (GFP-T1 and GFP-T2) with each of the Cas13 variants and measured the virus-expressed GFP signal 3 days post-infiltration. Notably, we observed a broad range in the interference levels between the different Cas13 variants. Our data show that both CasRx variants (CasRx-NLS and CasRx-NES) exhibited the highest level of interference against TRBO-GFP using GFP-T1 and GFP-T2 crRNAs compared to the other Cas13 orthologues. In addition, other variants including LwaCas13a-NLS, LwaCas13a-NES, and PspCas13p-NES exhibited an efficient interference against TRBO-GFP using also the two GFP-targeting crRNAs (Fig. 2a). In addition, as another control, we conducted one set of experiments in which we delivered the crRNAs and the target virus (TRBO-GFP) in the inoculated leaves, but without the Cas13 protein variants. We did not observe any virus interference, indicating that the virus interference observed in Fig. 2a is exclusively Cas13-crRNA dependent (Additional file 1: Figure S2). We repeated these transient assays in at least three independent experiments using the GFP signal as an indicator of RNA virus interference, and our data showed that CasRx is highly efficient compared to other Cas13 variants and NS-crRNA controls (Fig. 2b).

Next, we conducted molecular analyses to assess the RNA virus interference via immunoblotting of the GFP protein of TRBO-GFP virus to validate the observed reduction in the GFP signal. Our immunoblotting data corroborated our phenotypic data (GFP signal in inoculated leaves) and showed that LwaCas13a-NLS, LwaCas13a-NES, and PspCas13b-NES mediated strong interference against the RNA virus. Intriguingly, CasRx mediated the highest and most efficient interference when compared to these or other Cas13 variants, as indicated with the low GFP protein level of the targeted virus (Fig. 2c, d).

Because the CRISPR-Cas13 machinery targets the RNA transcripts of the virus for degradation, we wanted to assess the interference at the RNA level. Therefore, we conducted reverse transcription-quantitative PCR (RT-qPCR) to assess the interference activities of different Cas13 variants. Consistent with the level of GFP signal, the RT-qPCR data showed that CasRx mediated robust and highly efficient RNA interference when compared to other Cas13 variants (Fig. 2e). These data corroborated our phenotypic and immunoblotting data and identified CasRx as the most efficient Cas13 variant for RNA interference applications.

### CasRx outperforms LwaCas13a and PspCas13b variants in targeting the virus replicase for RNA interference

**Fig. 3:**
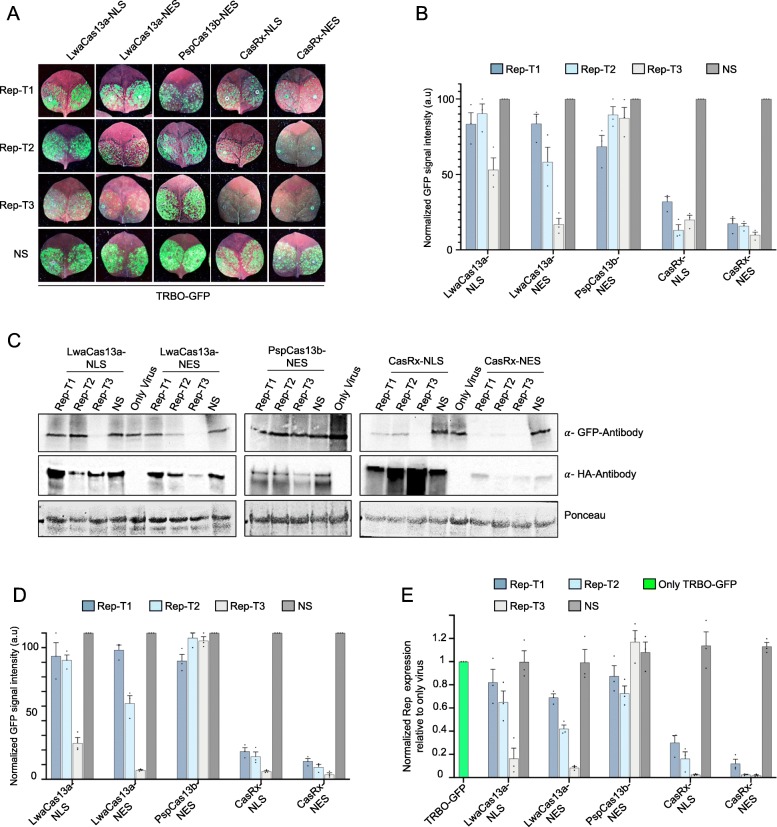
Characterization of LwaCas13a, PspCas13b, and CasRx activity against essential and conserved genomic region of RNA viruses. **a** GFP monitoring to assess the Cas13-mediated virus interference activities in *Agro*-infiltrated wild type *N. benthamiana* leaves in transient assays. Images were taken 3 days post-infiltration. NS, non-specific crRNA; Rep, replicase. **b** Relative fluorescence intensity quantification of leaf images. For each Cas13 variant, GFP signal intensity of each targeting crRNA (Rep-T1, Rep-T2, and Rep-T3) is shown relative to the non-targeting (NS) crRNA. Values shown as mean ± SEM (*n* = 3). **c** Western blot analysis of the abundance of the virus expressed GFP protein to confirm the Cas13-mediated TRBO-GFP virus interference. Protein blots were developed with anti-GFP and anti-HA antibodies. *α*-GFP, anti-GFP antibody, *α*-HA, anti-HA antibody. Ponceau staining served as loading control. **d** Quantification of the western blot data. For each Cas13 variant, the abundance of the GFP protein with the targeting crRNAs (Rep-T1, Rep-T2, and Rep-T3) is shown relative to the non-targeting (NS) crRNA. Error bars indicate SEM (*n* = 3). **e** RT-qPCR analysis of TRBO-GFP knockdown with different Cas13 variants using the three position-matched crRNAs. Transcript levels are shown relative to leaves inoculated with only TRBO-GFP vector. Values shown as mean ± SEM (*n* = 3)

In our previous interference experiments using crRNA against the GFP sequence of the virus genome, we identified three Cas13 orthologues with the highest level of interference activity against the TRBO-GFP RNA genome, namely LwaCas13a, PspCas13b, and CasRx. However, CasRx mediated the highest interference efficiency among these variants. Next, we wanted to test whether this interference activity and efficiency are applicable to other virus sequences and whether targeting other (conserved) virus sequences would result in similar interference efficiencies. Therefore, we designed three crRNAs targeting the replicase gene of the virus (Rep-T1, Rep-T2, and Rep-T3) and tested the activity of LwaCas13a, PspCas13b, and CasRx variants for RNA virus interference. Our data showed that CasRx mediated the highest level of interference activity, followed by LwaCas13a proteins (Fig. 3a). Interestingly, and despite its strong interference activity against the GFP sequence of the virus, PspCas13b did not mediate an efficient interference against the replicase transcripts using the three crRNAs (Fig. 3a). We also found that different crRNAs targeting different regions of the replicase transcripts exhibited variable interference efficiencies, where Rep-T3 crRNA mediated better interference relative to the other crRNAs (Fig. 3a), probably due to different factors affecting target accessibility [24]. Strikingly, CasRx mediated strong interference with all crRNAs used to target the replicase transcripts, with CasRx-NES mediating better interference compared to the CasRx-NLS variant (Fig. 3a). Independent repetition of these experiments against the virus replicase transcripts showed similar and consistent results, and confirmed that CasRx mediated robust RNA virus interference efficiencies when compared to other Cas13 variants (Fig. 3b).

Subsequently, we conducted molecular analyses using immunoblotting to assess the virus interference activities of these Cas13 variants. The immunoblotting data showed that targeting the Rep-T3 region within the virus replicase sequence provided strong interference with both LwaCas13a variants relative to the other two crRNAs, with improved efficiency with the cytoplasmic variant of LwaCas13a (LwaCas13a-NES) as evident with the Rep-T2 crRNA (Fig. 3c). Consistent with the phenotypic results of the GFP signal, no reduction in the GFP protein level was detected with the PspCas13b-NES protein in comparison to the NS control, with exception of the Rep-T1 crRNA that was better than other crRNAs (Fig. 3c). Analyzing the interference activities of CasRx showed that CasRx mediated highly efficient RNA interference using the three crRNAs targeting the replicase sequences. CasRx-NLS was efficient with the three crRNAs, but the highest efficiency was observed when Rep-T3 crRNA was used (Fig. 3c). Notably, CasRx-NES was efficient in conferring RNA virus interference against the RNA virus using all three crRNAs (Fig. 3c). Of note, although the protein levels of CasRx-NES were much lower compared to CasRx-NLS, the interference efficiencies of CasRx-NES were much stronger and more robust, indicating the importance of the localization of the Cas13 variant for virus interference activities (Fig. 3c). We repeated these experiments three times with similar results, and our combined data showed that CasRx mediated robust RNA virus interference using three crRNAs against the replicase transcripts (Fig. 3d).

To further validate these observations, RT-qPCR was performed to detect the accumulation of the TRBO-GFP genome. Consistent with the phenotypic and immunoblotting data, RT-qPCR results showed a significant reduction in the accumulation of the TRBO-GFP genomic RNA with Rep-T3 crRNA of both LwaCas13a and CasRx variants. Notably, CasRx was able to mediate efficient virus targeting with all three crRNAs in comparison with LwaCas13a or PspCas13b variants, with increased efficiency observed with the CasRx-NES variant (Fig. 3e). These data indicate that CasRx is capable of mediating robust and consistent efficiency in targeting different genomic sequences of the virus genome.

### CasRx confers highly efficient interference against TuMV

Next, we tested whether the interference activities of the Cas13 variants conducted in transient assays in inoculated leaves were similar when using a different virus capable of systemic spread throughout the plant. To this end, we conducted a set of transient assays in wild-type *N. benthamiana* plants using the TuMV-GFP virus as our target. We co-delivered the Cas13 variants (LwaCas13a-NLS, LwasCas13a-NES, PspCas13b-NES, CasRx-NLS, and CasRx-NES) identified from our previous experiments to confer strong RNA virus interference activities, with their cognate crRNAs targeting different genomic sequences of virus genome, and TuMV-GFP as a target. Cas13-mediated interference against the TuMV-GFP genome would result in attenuated replication and, thus, spread of the virus, which can be measured by monitoring the spread of the virus-expressed GFP to systemic leaves [33]. Our data showed that LwaCas13a-NES mediated strong interference against TuMV-GFP by limiting its systemic spread when HC-Pro and GFP-T2 crRNAs were used (Additional file 1: Figure S3). In addition, LwaCas13a-NLS mediated interference activities with the same crRNAs, but to a lower efficiency when compared to the LwaCas13a-NES variant. Moreover, PspCas13b-NES mediated modest interference when compared to LwaCas13a-NES with these position-matched crRNAs (Additional file 1: Figure S3). The interference activities were variable based on the crRNA that was used, with HC-Pro and GFP-T2 crRNAs providing the highest activity in conferring virus interference. Relative to the other tested Cas13 variants, CasRx exhibited the strongest and most efficient interference activities with CasRx-NLS and CasRx-NES using all crRNAs (Additional file 1: Figure S3). However, CP crRNA was the least efficient when compared to other crRNAs, and CasRx-NES was better than CasRx-NLS with all crRNAs tested, corroborating our previous data and indicating that CasRx was the most efficient Cas13 variant for RNA virus interference (Additional file 1: Figure S3).

**Fig. 4:**
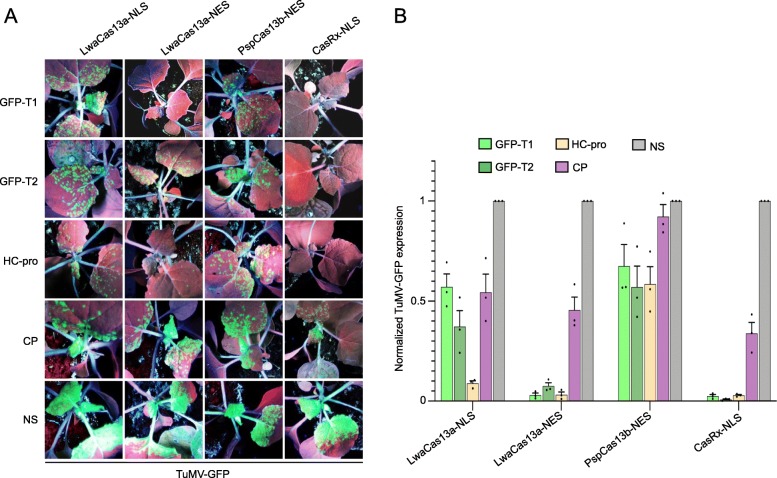
CasRx mediates efficient interference against TuMV-GFP virus by preventing its systemic spread. **a** Cas13-mediated interference against the GFP-expressing TuMV virus in plants. *N. benthamiana* plants expressing LwaCas13a, PspCas13b, and CasRx proteins were co-infiltrated with TRV (expressing crRNAs targeting the TuMV-GFP virus and NS crRNA) and TuMV-GFP. At 7 dpi, plants were imaged under UV light for GFP signal monitoring. **b** RT-qPCR analysis of TuMV-GFP knockdown with different Cas13 variants using the different position-matched crRNAs. For each Cas13 variant, knockdown efficiency of each targeting crRNA is shown relative to the non-targeting (NS) crRNA. Values shown as mean ± SEM (*n* = 3)

Next, we tested whether these interference activities would be observed in plants stably expressing Cas13 variants, compared to the transient expression experiments performed above. To this end, we generated stable expression lines of *N. benthamiana* plants expressing Cas13 variants, including LwaCas13a-NLS, LwaCas13a-NES, PspCas13b-NES, and CasRx-NLS, using *Agrobacterium tumefaciens*-mediated transformation to assess the interference efficiencies of these Cas13 variants. We confirmed the expression of Cas13 proteins in the generated permanent lines, and our western blotting analysis show that the different Cas13 proteins were expressed efficiently in *N. benthamiana* plants, and the correct proteins sizes were detected (Additional file 1: Figure S4). Using Cas13 overexpressing lines with relatively similar level of protein expression, we conducted our RNA interference experiments using TuMV-GFP as the target virus and delivered different crRNAs (GFP-T1, GFP-T2, HC-Pro, and CP, as well as NS-crRNA) via the TRV system. All these Cas13 variants exhibited RNA virus interference, but with different efficiencies among the different crRNAs (Fig. 4a). Consistent with our previous data, LwaCas13a-NES was better with all crRNA tested than LwaCas13a-NLS, and both of these variants exhibited better virus interference than PspCas13b-NES (Fig. 4a). Most importantly, we found that the CasRx variant resulted in the highest level of virus interference using different crRNAs compared to other Cas13 variants (Fig. 4a).

We next investigated the virus interference activities of these Cas13 variants at the molecular level. Therefore, we conducted RT-qPCR assays to quantify the RNA message of the virus in the systemic leaves of the infected plants of different Cas13 variants and crRNAs. Consistent with the TuMV-GFP signal observed in the phenotypic data, our RT-qPCR results showed that CasRx mediated the highest interference efficiency in conferring RNA virus interference across all crRNAs used compared to other variants, with comparable knockdown efficiency of GFP-T1 crRNA between LwaCas13a-NES and CasRx-NLS variants (Fig. 4b). Therefore, we concluded that CasRx conferred much higher efficiency interference compared to other Cas13 variants.

### CasRx catalytic activity is required for RNA virus interference

**Fig. 5:**
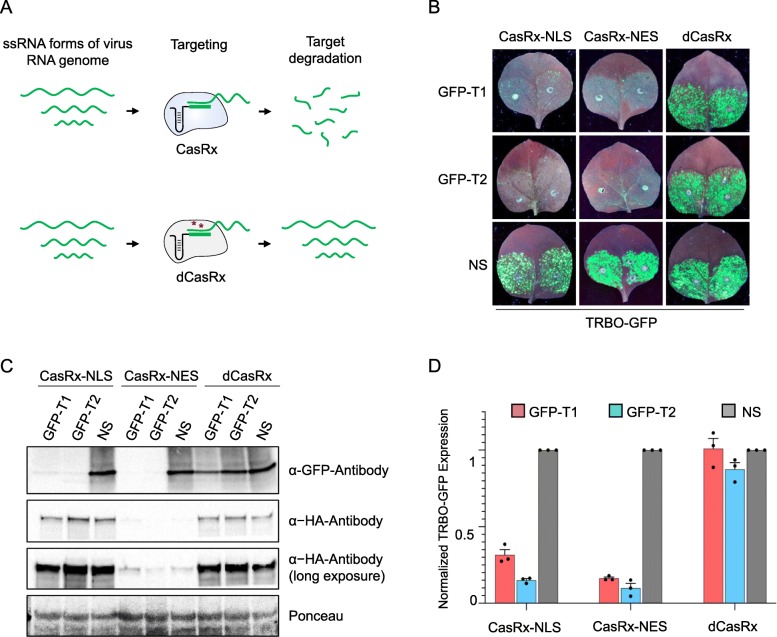
CasRx catalytic activity is required for RNA virus interference. **a** Illustration of the targeting activity of the catalytically active and catalytically deactivated (dCasRx) CasRx variants against the targeted TRBO-GFP virus. **b** GFP monitoring to assess the Cas13-mediated virus interference activities in *Agro*-infiltrated wild type *N. benthamiana* leaves in transient assays. Images were taken 3 days post-infiltration. NS, non-specific crRNA. **c** Western blot analysis of the abundance of the virus expressed GFP protein to confirm the Cas13-mediated TRBO-GFP virus interference. Protein blots were developed with anti-GFP and anti-HA antibodies. *α*-GFP, anti-GFP antibody, *α*-HA, anti-HA antibody. Ponceau staining served as loading control. **d** RT-qPCR analysis of TRBO-GFP knockdown with the catalytically active and catalytically deactivated CasRx variants. For each CasRx variant, knockdown efficiency of each targeting crRNA (GFP-T1 and GFP-T2) is shown relative to the non-targeting (NS) crRNA. Values shown as mean ± SEM (*n* = 3)

Our RNA virus interference transient and stable overexpression assays indicated that CasRx mediated high efficient virus interference with different crRNAs targeting TRBO-GFP or TuMV-GFP viruses. We wanted to investigate whether the catalytic activity of CasRx is required to confer interference against RNA viruses. It is possible that binding of CasRx to the virus genome may attenuate its replication and translation, leading to interference activities mediated by binding, but not the catalytic degradation of the RNA transcripts [45]. Therefore, we used a catalytically deactivated CasRx (dCasRx) variant harboring mutations in the catalytic HEPN domains of the CasRx-NLS protein (R239A/H244A/R858A/H863A). These mutations have been reported to abrogate the catalytic cleavage activity but not the binding activity of the CasRx protein [40]. Subsequently, we co-delivered the catalytically active variants of CasRx (CasRx-NLS and CasRx-NES) and the catalytically deactivated variant of CasRx (dCasRx) into the leaves of *N. benthamiana* plants with TRBO-GFP virus as a target as well as GFP-T1, GFP-T2, and NS crRNAs. Our data revealed that only the catalytically active variants of CasRx mediated virus interference and that the dCasRx variant did not show any interference activities (Fig. 5a, b).

To validate this observation, we conducted immunoblotting and RT-qPCR assays to detect and quantify the virus-expressed GFP protein as well as the accumulation of the virus RNA genome with either the catalytically active CasRx-NLS and CasRx-NES variants or the catalytically deactivated (dCasRx) variant. Consistent with the phenotypic data, we observed a significant reduction in the abundance of the virus GFP protein with the catalytically active CasRx variants compared to the dCasRx (Fig. 5c). In addition, a significant knockdown of the targeted RNA virus was observed with the catalytically active CasRx variants with both crRNAs, while no decrease in the RNA level of the targeted TRBO-GFP virus genome was observed with dCasRx (Fig. 5d). These data indicated that CasRx-mediated virus interference is dependent on the catalytic activity of CasRx and suggesting that binding of dCasRx to the virus genome does not inhibit its replication.

### CasRx exhibits multiplexed and specific virus-targeting activity with no observed collateral activity

**Fig. 6:**
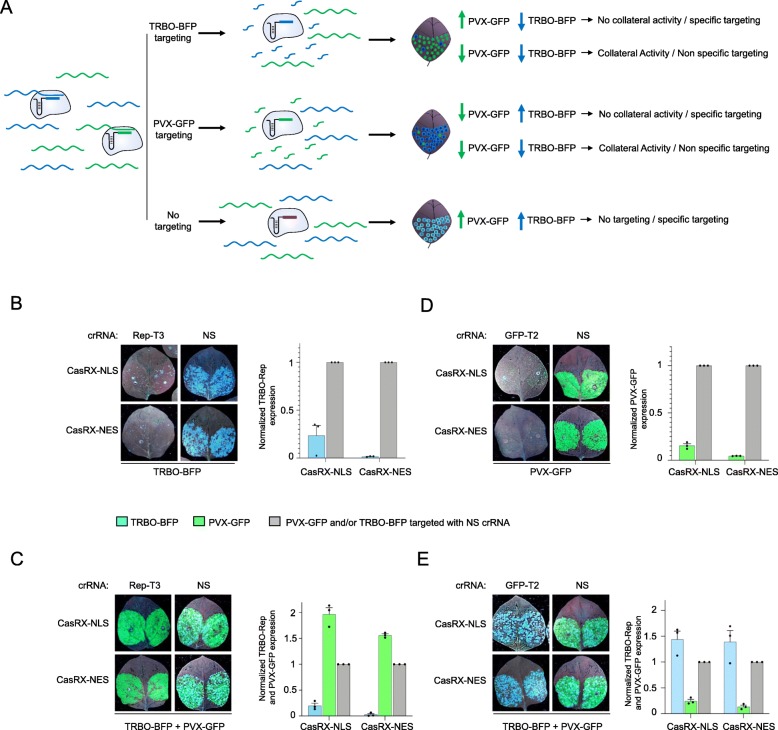
Specific virus targeting via CasRx with no observed collateral activity. **a** Schematic of CasRx targeting of TRBO-BFP and PVX-GFP viruses in synchronous co-infected leaves with the two different viral vectors, and the possible outcomes. **b** BFP monitoring to assess the Cas13-mediated virus interference activities in *Agro*-infiltrated wild type *N. benthamiana* leaves in transient assays (left). Images were taken 3 days post-infiltration. NS, non-specific crRNA; Rep, replicase. RT-qPCR analysis of TRBO-BFP knockdown with Rep-T3 crRNA used against TRBO-BFP virus (right). The transcript levels of TRBO-BFP are shown relative to the transcript level of TRBO-BFP virus targeted with (NS) crRNA. Values shown as mean ± SEM (*n* = 3). **c** BFP and GFP monitoring to assess the Cas13-mediated virus interference activities in *Agro*-infiltrated wild type *N. benthamiana* leaves in transient assays (left). Images were taken 3 days post-infiltration. NS, non-specific crRNA; Rep, replicase. RT-qPCR analysis of TRBO-BFP and PVX-GFP knockdown with Rep-T3 crRNA (right). The transcript levels of TRBO-BFP are shown relative to the transcript level of the non-targeted virus (PVX-GFP), and the transcript level of both viruses is shown relative to the transcript level of both viruses targeted with (NS) crRNA. Values shown as mean ± SEM (*n* = 3). **d** GFP monitoring to assess the Cas13-mediated virus interference activities in *Agro*-infiltrated wild type *N. benthamiana* leaves in transient assays (left). Images were taken 3 days post-infiltration. NS, non-specific crRNA. RT-qPCR analysis of PVX-GFP knockdown with GFP-T2 crRNA used against PVX-GFP virus (right). The transcript levels of PVX-GFP are shown relative to the transcript level of PVX-GFP virus targeted with (NS) crRNA. Values shown as mean ± SEM (*n* = 3). **e** BFP and GFP monitoring to assess the Cas13-mediated virus interference activities in *Agro*-infiltrated wild type *N. benthamiana* leaves in transient assays (left). Images were taken 3 days post-infiltration. NS, non-specific crRNA. RT-qPCR analysis of TRBO-BFP and PVX-GFP knockdown with GFP-T2 crRNA (right). The transcript levels of PVX-GFP are shown relative to the transcript level of the non-targeted virus (TRBO-BFP), and the transcript level of both viruses is shown relative to the transcript level of both viruses targeted with (NS) crRNA. Values shown as mean ± SEM (*n* = 3)

We have shown that CasRx is a robust Cas13 variant for RNA virus interference applications *in planta*. Specificity of CasRx is an important issue in its interference applications against viruses. Therefore, we sought to test the specificity of CasRx against different RNA viruses. We developed a virus-based reporter system that relies on synchronous coinfection and co-replication of two different viral vectors. The two vectors need to be derived from different RNA viruses so they can non-competitively replicate and exist in the same cell [46]. Therefore, besides our TRBO RNA virus, we adopted chimeric *potato virus X* expressing GFP (PVX-GFP), which has been found to be non-competing with the TMV-based vectors [46]. To build an easy-to-monitor system to determine CasRx specificity, we replaced the GFP sequence in our TRBO-GFP chimeric virus with an enhanced blue fluorescent protein (EBFP)-encoding sequence, resulting in the generation of a TRBO-BFP construct. We tested the engineered TRBO-BFP construct and detected a blue fluorescent signal instead of the GFP signal, indicating that our modification was successful. Subsequently, we co-delivered CasRx-NES and CasRx-NLS variants, PVX-GFP and TRBO-BFP, into the leaves of *N. benthamiana* with crRNAs providing specificity to either virus. Theoretically, by delivering virus-specific crRNAs, only the titer of the targeted virus will be mitigated, indicating specific interference against the targeted virus. However, if the interference activity is not specific, interference of both viruses will occur (Fig. 6a). Our data showed that using CasRx variants to target the TRBO-BFP virus, the interference activity occurred against the TRBO-BFP virus alone, but the co-delivered PVX-GFP signal was high, indicating the absence of interference activity against the non-targeted virus (Fig. 6b, c). Similarly, when we targeted the PVX-GFP virus, only the GFP signal was mitigated, and not the BFP signal generated by TRBO-BFP (Fig. 6d, e). Quantification of the viruses’ genomic RNA by RT-qPCR showed robust knockdown of the targeted viruses, whereas no change in the abundance of the non-targeted virus or viruses with non-specific (NS) crRNA was observed, indicating the specificity of the targeted virus interference (Fig. 6). Next, we assessed the capability of CasRx to mediate multiplexed knockdown of different RNA viruses. Therefore, we co-delivered each of the catalytically active CasRx variants with both PVX-GFP and TRBO-BFP viruses into the leaves of *N. benthamiana* with simultaneous delivery of two different crRNAs, each providing specificity to either virus (GFP-T2 against PVX-GFP, and Rep-T3 against TRBO-BFP virus). Our data show that both CasRx variants were able to exhibit efficient targeting of both viruses simultaneously using the two (GFP-T2 and Rep-T3) crRNAs, while no virus interference was observed when NS crRNA was used (Additional file 1: Figure S5a, S5b). These results indicate the high specificity of CasRx in targeting RNA viruses, and that CasRx is amenable to multiplex targeting of different RNA viruses in plants.

## Discussion

Prokaryotic adaptive immunity systems have been harnessed for engineering genomes and transcriptomes across eukaryotic species [47]. Different class II type VI CRISPR-Cas13 systems have been employed for transcriptome engineering and nucleic acid detection in mammalian cells. Despite the fact that several Cas13 variants have been reported with variable crRNA scaffolds, sizes, and importantly catalytic activities, CRISPR-LshCas13a has been the only Cas13 system used for RNA virus interference activities in plants. Here, we characterized different Cas13 variants for robust interference against RNA viruses in transient assays and stable *N. benthamiana* Cas13-overexpression plants. We employed RNA virus interference activity reporter systems and conducted a comprehensive screen to identify the most catalytically active Cas13 variant in conferring RNA interference. Our data revealed that CasRx exhibits the most robust catalytic activity among the variants tested.

In this study, we constructed different Cas13 variants, including Cas13a (LshCas13a and LwaCas13a), Cas13b (PspCas13b and BzCas13b), and Cas13d (CasRx), and their corresponding crRNA scaffolds for *in planta* expression. Transient assays using TRBO-GFP as the interference target showed that CasRx was the most efficient in conferring interference using GFP-T1 and GFP-T2 crRNAs. In addition, LwaCas13a and PspCas13b exhibited strong efficiencies compared to LshCas13a and BzCas13b variants. Interestingly, although we used position-matched crRNAs with all the tested Cas13 proteins, these crRNAs exhibited variable efficiencies among the Cas13 variants. This might result from the diverse molecular behaviors, molecular architectures, and protospacer flanking sequence (PFS) requirements for these different Cas13 variants [48]. We selected the top three variants for further analysis and tested them in targeting a conserved virus sequence of the replicase transcript of TMV. Our data showed that CasRx exhibited robust virus interference using the three crRNAs that were designed against the replicase sequence. However, other variants exhibited interference, and LwaCas13a was more efficient than PspCas13b. The fact that CasRx targeted the different regions of the replicase sequence with high efficiency indicates the competency and suitability of this system for efficient targeting of other essential sequences in other RNA viruses, and offers the potential development of this system for further RNA virus targeting and manipulation. Molecular analysis corroborated our phenotypic data and indicated that in general, cytoplasmic-localized Cas13 (Cas13-NES) provided better interference than the NLS-localized variants (Cas13-NLS), which could be explained by the simultaneous localization of the cytoplasmic-localized Cas13 proteins and the targeted virus in the plant cytoplasm, where the targeted viruses replicate.

To test whether these variants limit systemic spread of the virus with similar efficiencies, we used TuMV-GFP as a target and co-delivered the Cas13 variants with their corresponding crRNAs. CasRx provided robust interference against the TuMV virus, thus significantly limited the spread of the TuMV-GFP virus into systemic leaves. In addition, the cytoplasmic-localized LwaCas13a provided strong interference compared to PspCas13b. To corroborate these data, we generated *N. benthamiana* overexpression lines of these Cas13 variants and conducted interference assays. Our data showed that CasRx is indeed the most robust Cas13 variant to confer virus interference followed by the LwaCas13a-NES variant. These data indicate that CasRx is an excellent candidate for RNA virus interference applications in plants.

Previous studies harnessing CRISPR-Cas proteins, such as FnCas9 from *Francisella novicia*, to target RNA viruses have shown that the binding, but not the cleavage, ability of these proteins is sufficient to inhibit virus replication in mammalian and plant cells [45, 49]. Our data show that the HEPN-dependent catalytic activity of CasRx is essential for efficient virus interference, which in turn indicate that the observed CasRx-mediated virus interference results from targeted viral genomic degradation. However, the ability of targeting the dCasRx to specifically bind to mRNA transcripts may have various uses in RNA transcript manipulations. For example, fusion of dCasRx to a splicing effector has been used to manipulate alternative splicing in mammalian cells [40]. Similarly, development of dCasRx as a specific and programmable RNA-binding platform in plants would offer various applications, including virus transcript tracking and imaging to study the biology of virus replication. Furthermore, the generation of chimeric fusions of dCasRx and other functional domains may be beneficial for developing CasRx-based regulatory reagents for imaging of endogenous mRNA and non-coding RNA, fine-tuning and regulation of alternative splicing, tracking and trafficking, translational control, and regulation under diverse growth, environmental, and developmental conditions and cues [50, 51].

The specificity of CasRx for in vivo application is an important point to consider. Therefore, we evaluated the specificity of CasRx in the context of virus targeting *in planta*, and our data show that the CasRx targeting activity is highly specific to the targeted virus, where the transcript abundance of the co-delivered, non-targeted, virus was not affected. Cas13 variants possess robust collateral activities in vitro, enabling the development of pathogen detection systems that have revolutionized molecular diagnostics [52, 53]. Although the collateral activities of Cas13 variants are an essential part of the broader immunity conferred by CRISPR-Cas13 systems in prokaryotes [25], such activity is not observed in human and plant cells [33, 38]. Here, we could infer from these results that CasRx, and probably other Cas13 variants, lack such collateral activity in plants. Nevertheless, further experiments and analysis are needed to corroborate this conclusion. It remains to be tested whether accessory proteins, including CXS28 and WYL, can stimulate the activity of CasRx against the targeted transcripts and induce collateral activities *in planta*. This interference system can further be improved by determining the molecular basis of how the type VI system mediates antiviral defenses within their own hosts (spacer acquisition and adaptation) and how these effectors might function in eukaryotic hosts. These questions warrant detailed studies to understand the molecular functions, and the basis of the catalytic activities, of these proteins for broader applications in endowing immunity to eukaryotes.

In addition, we have shown that CasRx can be used to target either one virus alone or two RNA viruses simultaneously, thereby extending the applicability of this interference system. Such system might have the potential use in crop species for virus interference of mixed infections of RNA viruses that occur in the field and under natural conditions. It is worth noting that recent studies have shown the ability of DNA viruses to evolve as a result of Cas9 targeting [21, 54]. Therefore, further studies are needed to test the ability of RNA viruses to evolve or escape the CRISPR Cas13 machinery in plants. Interestingly, while preparing this manuscript, a recent study harnessed different CRISPR-Cas13 systems, including LwaCas13a and PspCas13b, for interference against several human RNA viruses. Importantly, the study showed that Cas13 targeting of the virus genome does not result in mutations at the crRNA target sites, suggesting that viruses targeted by CRISPR-Cas13 systems may not be able to evolve resistance to CRISPR-Cas13 machinery [55].

## Conclusion

In conclusion, CRISPR-CasRx enables programmable RNA virus interference in plants and has the potential to be optimized and developed in the future for transcriptome engineering applications. CRISPR-CasRx expands the power of CRISPR systems for virus interference applications and may help to address basic plant virology questions.

## Methods

### Vector construction

#### Construction of Cas13 orthologues for *in planta* expression

Nine different Cas13 variants were constructed for activity screening in plants and were cloned in binary vectors for *in planta* expression. The DNA sequences of the LshCas13a variants were plant codon optimized and subsequently cloned into binary vectors, as described previously [33]. Similarly, plant codon-optimized versions of BzCas13b variants with fusions of the 3xHA tag at the N terminus were designed and synthesized (Blue Heron Biotech), either with or without the NLS-encoding sequence at the C terminus, and were flanked by attL1 and attL2 sites to facilitate Gateway cloning into binary vectors (Additional file 1: Sequence S1, S2). Subsequently, BzCas13b-NLS and BzCas13b (no NLS) were subcloned into *pK2GW7* binary vectors using LR Gateway recombination cloning to generate *pK2GW7*-BzCas13b, in which expression is controlled by the constitutive 35S cauliflower mosaic virus promoter (Additional file 1: Map S1). For the other Cas13 orthologues, including LwaCas13a-NLS/NES, PspCas13b-NES, CasRx-NLS, and dCasRx-NLS, the DNA sequences were amplified from plasmids provided by Addgene (Addgene plasmid numbers 91902/105815, 103862, 109049, and 109050, respectively) using primers that add a CACC sequence to the 5′ end of the amplified sequence to facilitate subsequent directional cloning into pENTR/D-TOPO vectors (Invitrogen) (Additional file 1: Table S2). Subsequently, Cas13 sequences were transferred from the pENTR/D-TOPO entry vectors into *pK2GW7* binary vectors using Gateway recombination reactions. To generate the CasRx-NES version, the CasRx ORF sequence was amplified from the CasRx-NLS vector (plasmid number 109049) using primers that remove NLS sequences from the N and C termini of CasRx-NLS ORF, leading to the removal of the HA tag and GFP-encoding sequences, and BsaI sites were added at both ends of the isolated ORF (Additional file 1: Table S2). To add nuclear export sequences (NES), DNA sequences encoding NES and NES-1xHA tags were purchased as single-stranded DNA (ssDNA) oligonucleotides from Sigma with overhangs complementary to the overhangs generated from digestion of the BsaI sites at the two ends of the isolated CasRx ORF sequence (Additional file 1: Table S2). Phosphorylated and annealed double-stranded DNA (dsDNA) oligonucleotides were ligated into the BsaI-digested DNA sequence of the CasRx ORF to produce the 5′-NES-CasRx-NES-HA-3′ sequence. Subsequently, the ligation product of 5′-NES-CasRx-NES-HA-3′ was PCR amplified and subcloned into the pENTR/D-TOPO vector, followed by transfer into the *pK2GW7* binary vector using LR Gateway recombination cloning. All the Cas13 variant sequences were confirmed in *pK2GW7* binary vectors with Sanger sequencing.

#### Generation of the TRBO-BFP construct

To generate the TRBO infectious clone expressing BFP, the EBFP-encoding sequence was amplified from the LeGO-EBFP2 vector (Addgene plasmid number 85213) with primers that add AseI and NotI restriction sites to the 5′ and 3′ ends of the amplified BFP sequence, respectively (Additional file 1: Table S2). The GFP-encoding sequence was removed from the pJL-TRBO-G clone (Addgene plasmid number 80083) by digestion with AseI and NotI and replaced by the ligation of the AseI- and NotI-digested PCR product of BFP into the digested vector.

#### Construction of crRNA expression constructs

To construct the crRNA expression clones, the orthologue’s corresponding direct repeat with the targeting or non-targeting (NS) sequences was purchased from Sigma as ssDNA oligonucleotides (all crRNA sequences used in this study are listed in Additional file 1: Table S1). The phosphorylated and annealed dsDNA fragments of crRNAs were ligated into the TRV RNA2 genome under the pPEBV promoter using XbaI and either XhoI or BamHI restriction sites.

### Plant material

Two- to 3-week-old wild-type *Nicotiana benthamiana* plants grown under long-day conditions (16 h light, 8 h dark at 25 °C) were used for all transient experiments. To generate transgenic *Nicotiana benthamiana* plants overexpressing different Cas13 orthologues, *Agrobacterium tumefaciens* GV310 strains harboring the selected constructs were transformed into *N. benthamiana* leaf discs following previous published protocols [56]. Generated plants (T1) were screened for the expression of the Cas13 protein by immunoblotting using an anti-HA antibody, and seeds harvested from the Cas13-positive plants were screened on kanamycin-containing MS (Murashige and Skoog) media. Surviving seedlings were used for virus-targeting experiments.

### Agroinfiltration of *N. benthamiana* leaves

The different constructs (including all *pK2GW7*-Cas13 constructs, the TRV RNA1 genome, the engineered TRV RNA2 genomes harboring crRNA sequences under the pPEBV promoter, and the different infectious clones of the RNA viruses, including TRBO-GFP, TRBO-BFP, PVX-GFP, or TuMV-GFP) were individually electroporated into *A. tumefaciens* strain GV3101. Single colonies grown overnight in selective medium were centrifuged and suspended in infiltration medium (10 mM MES [pH 5.7], 10 mM MgCl_2_, and 200 μM acetosyringone) and incubated at ambient temperature for 2 h. For infiltration into leaves of *N. benthamiana* plants, cultures were mixed at a final OD_600_ of 0.5 for *pK2GW7*-Cas13, 0.1 for RNA1 and RNA2-crRNAs, 0.05 for TuMV-GFP and PVX-GFP, and 0.005 for TRBO-GFP and TRBO-BFP, relative to the suspended culture grown overnight. Healthy and fully developed leaves were selected for experiments, and agroinfiltration was done with a 1-ml needleless syringe.

### Fluorescent protein intensity assays and quantitative fluorescence intensity analysis of images

For GFP and/or BFP expression in transient studies, infiltrated leaves were cut and then photographed after 3 days under a handheld UV light in the dark. For the systemic TuMV-expressed GFP in wild-type and transgenic plants, the whole plants were photographed after 6 to 7 days post-infiltration (dpi). For the quantitative measurements of the GFP signal intensity in images, the mean pixel values of the TRBO-GFP gene expression images were analyzed with ImageJ software to estimate GFP expression across samples. For each sample (each target of Cas13 variants), three biological replicates, represented as three different infiltrated leaves from three different plants, were analyzed. For each leaf, the GFP signal intensities were obtained as the corrected total cell fluorescence (CTCF) following this formula: CTCF = integrated density − (area of selected cell × mean fluorescence of background readings). The CTCF values of all three biological replicates for each sample were then averaged, and the normalized average of each target was compared to the normalized average CTCF value of the NS target. All values were limited to max = 100.

### Immunoblot analysis

Total proteins were extracted from 100 mg of sample using extraction buffer (100 mM Tris-Cl, pH 8, 150 mM NaCl, 0.6% IGEPAL, 1 mM EDTA, and 3 mM DTT) with protease inhibitors (PMSF, leupeptin, aprotinin, pepstatin, antipain, chymostatin, Na_2_VO_3_, NaF, MG132, and MG115). Proteins were separated on a 10% polyacrylamide gel. Immunoblot analysis was conducted using mouse α-GFP antibody (1:3000, Invitrogen) for virus-expressed GFP and rat α-HA (1:1000) antibody for detection of Cas13 proteins. The antigens were detected by chemiluminescence using an ECL-detecting reagent (Thermo Scientific). The quantitative graphs summarizing the average of three independent biological replicates were produced by calculating the densiometric data generated by the relative quantification of protein bands from immunoblot membranes using ImageJ software. The average data of three independent immunoblot replicates were calculated as previously described [57]. Results were presented as fold change relative to the NS and/or virus-only control samples.

### RNA extraction and RT-qPCR for analysis of viral RNA genomes

Total RNA was extracted from infiltrated leaves (in transient assays) or systemic leaves (in Cas13 overexpression lines) using Direct-zol RNA Miniprep Kits (Zymo Research) following the manufacturer’s instructions. Viral RNA was quantified with one-step RT-qPCR using the iTaq Universal SYBR Green One-Step Kit (Bio-Rad). All RT-qPCR reactions were performed in 10 μl reactions with three technical replicates in either 384-well or 96-well format and read out using a StepOnePlus™ Real-Time PCR System (Applied Biosystem) for the 96-well plates or the CFX384 Touch™ Real-Time PCR detection system (Bio Rad) for the 384-well plates. Expression levels were calculated by subtracting the housekeeping reference gene (tobacco *PP2A* [58]) cycle threshold (*C*_t_) values from target *C*_t_ values to normalize for total input, resulting in Δ*C*_t_ levels. Relative transcript abundance was computed as 2^−ΔΔ*C*t^ [59]. All samples were performed as three biological replicates.

## Supplementary information

**Additional file 1: Figure S1.** TRBO-GFP virus genome. **Figure S2.** Cas13 proteins are required for RNA virus interference. **Figure S3.** CasRx mediates efficient interference against TuMV-GFP virus by preventing its systemic spread in wild type *N. benthamiana* plants. **Figure S4.** Confirmation of Cas13 protein expression in permanent *N. benthamiana* lines. **Figure S5.** Multiplexed targeting of TRBO-BFP and PVX-GFP viruses through delivery of two different crRNAs with CasRx. **Sequence S1.** BzCas13b amino acid sequence (3xHA-BzCas13b-NLS). **Sequence S2.** BzCas13b full-length plant codon optimized DNA sequence (3x-HA-BzCas13b-NLS). **Map S1.** BzCas13b in *pK2GW7* (HA-BzCas13b-NLS). **Table S1.** crRNA sequences used in this study. **Table S2.** primers used in this study.

**Additional file 2.** Review history.

## Data Availability

Key clones used in this study are available from the Laboratory of Genome Engineering and Synthetic Biology at KAUST.
