# Supplementary material for: CRISPR-Cas13d mediates robust RNA virus interference in plants
Source: Genome Biol. 2019 Dec 2;20:263. doi: 10.1186/s13059-019-1881-2 (PMC6886189; doi:10.1186/s13059-019-1881-2)
Supplement: Supplementary file 1 — Additional file 1: Figure S1. TRBO-GFP virus genome. Figure S2. Cas13 proteins are required for RNA virus interference. Figure S3. CasRx mediates efficient interference against TuMV-GFP virus by preventing its systemic spread in wild type N. benthamiana plants. Figure S4. Confirmation of Cas13 protein expression in permanent N. benthamiana lines. Figure S5. Multiplexed targeting of TRBO-BFP and PVX-GFP viruses through delivery of two different crRNAs with CasRx. Sequence S1. BzCas13b amino acid sequence (3xHA-BzCas13b-NLS). Sequence S2. BzCas13b full-length plant codon optimized DNA sequence (3x-HA-BzCas13b-NLS). Map S1. BzCas13b in pK2GW7 (HA-BzCas13b-NLS). Table S1. crRNA sequences used in this study. Table S2. primers used in this study. [file 13059_2019_1881_MOESM1_ESM.docx]

**ADDITIONAL FILE 1**

**CRISPR-Cas13d mediates robust RNA virus interference in plants**

Ahmed Mahas^1, 2^, Rashid Aman^1, 2^, and Magdy Mahfouz^1,^*

*^1^Laboratory for Genome Engineering and Synthetic Biology, Division of Biological Sciences, 4700 King Abdullah University of Science and Technology, Thuwal 23955-6900, Saudi Arabia.*

*Correspondence: Magdy M. Mahfouz (magdy.mahfouz@kaust.edu.sa)

^2^These authors contributed equally to this work

**Key words:** CRISPR-Cas, Cas13, RNA interference, virus interference, virus resistance, CasRx

**Additional file 1:**

Figure S1: TRBO-GFP virus genome

Figure S2: Cas13 proteins are required for RNA virus interference.

Figure S3: CasRx mediates efficient interference against TuMV-GFP virus by preventing its systemic spread in wild type *N. benthamiana* plants

Figure S4: Confirmation of Cas13 protein expression in permanent *N. benthamiana* lines

Figure S5: Multiplexed targeting of TRBO-BFP and PVX-GFP viruses through delivery of two different crRNAs with CasRx

Sequence S1: BzCas13b amino acid sequence (3xHA-BzCas13b-NLS)

Sequence S2**:** BzCas13b full-length plant codon optimized DNA sequence (3x-HA-BzCas13b-NLS)

Map S1: BzCas13b in *pK2GW7* (HA-BzCas13b-NLS)

Table S1: crRNA sequences used in this study

Table S2: primers used in this study

GFP-T1

GFP-T2

3’ UTR

5’ UTR

**RdRp**

**MP**

**GFP**

ORF2

Rep-T3

Rep-T1

Rep-T2

**Figure S1: TRBO-GFP virus genome.**

Schematic representation of the TRBO-GFP virus genome with the targeted regions indicated.

**Figure S2: Cas13 proteins are required for RNA virus interference.**

1. Illustration of the activity of the crRNA against the targeted TRBO-GFP virus without Cas13 protein.
2. GFP monitoring to assess the possibility of crRNA-mediated virus interference activities in *Agro*-infiltrated wild type *N. benthamiana* leaves in transient assays. Images were taken 3 days post infiltration. NS: non-specific crRNA. 1: crRNAs for LshCas13a, 2: crRNAs for LwaCas13a, 3: crRNAs for BzCas13b, 4: crRNAs for PspCas13b, 5: crRNAs for CasRx
3. Western blot analysis of the abundance of the virus expressed GFP protein to confirm the crRNA-mediated TRBO-GFP virus interference. Protein blots were developed with anti-GFP antibody. $\alpha$-GFP. Ponceau staining served as loading control. 1, 2, 3, 4 and 5 indicate labelling as in B.
4. RT-qPCR analysis of TRBO-GFP transcript abundance in the leaves shown in B. For each set of crRNAs of each variant, knockdown efficiency of each targeting crRNA (GFP-T1 and GFP-T2) are shown relative to the non-targeting (NS) crRNA. 1, 2, 3, 4 and 5 indicate labelling as in B. Values shown as mean ± s.e.m. (n=3).


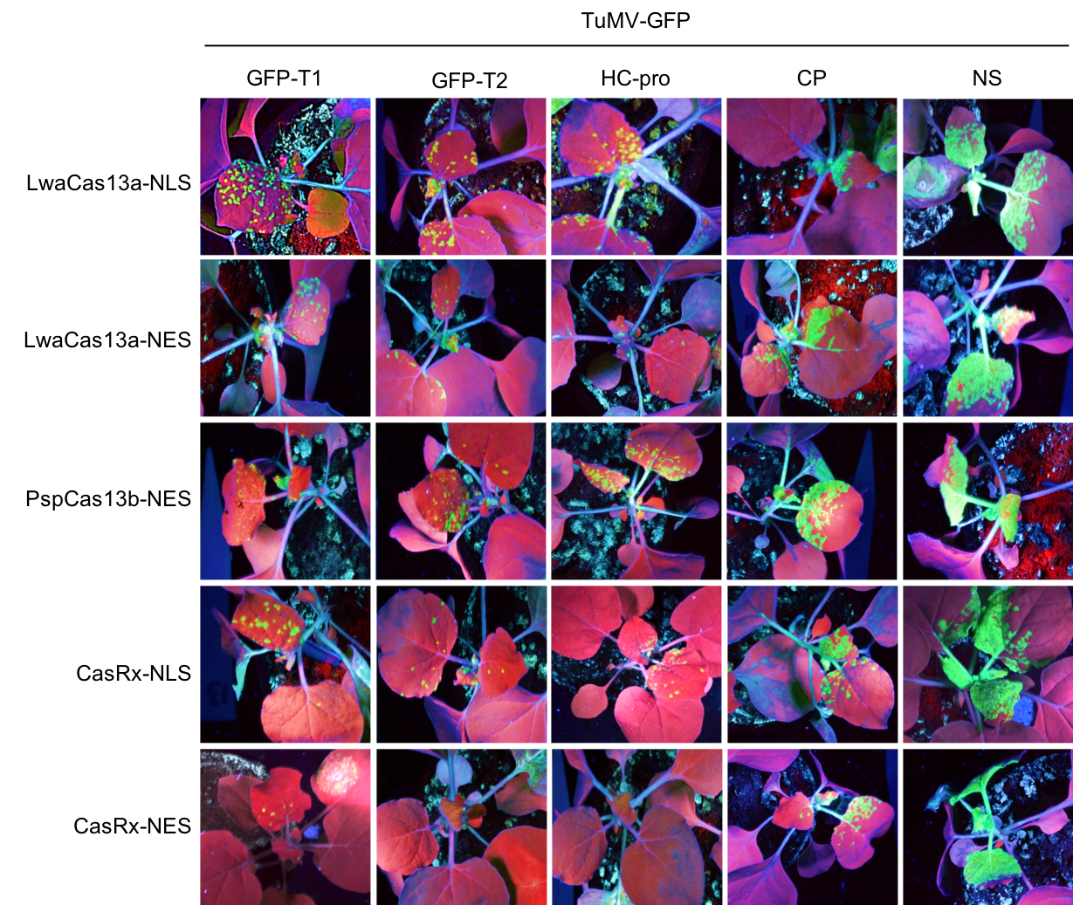


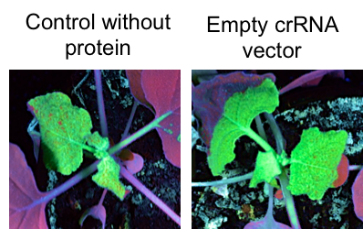


**Figure S3: CasRx mediates efficient interference against TuMV-GFP virus by preventing its systemic spread in wild type *N. benthamiana* plants.**

Wild type *N. benthamiana* plants were co-infiltrated with either LwaCas13a, PspCas13b, or CasRx variants and TRV (expressing crRNAs targeting the TuMV-GFP virus and non-specific (NS) crRNA) and TuMV-GFP. At 7 dpi, plants were imaged under UV light for GFP signal monitoring.

A


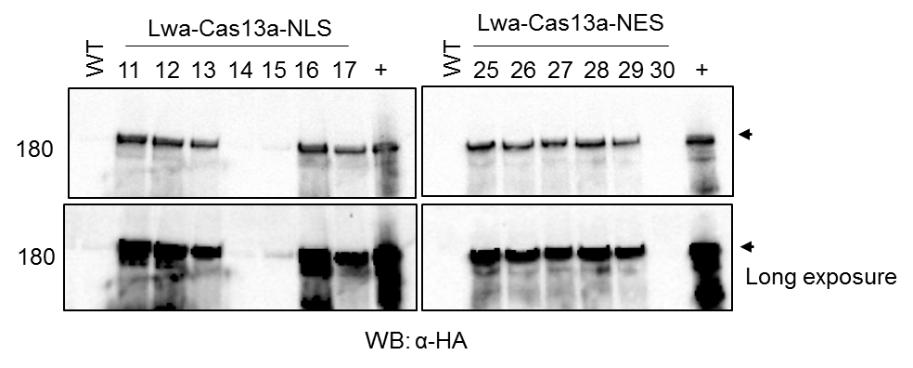


LwaCas13a

B

C


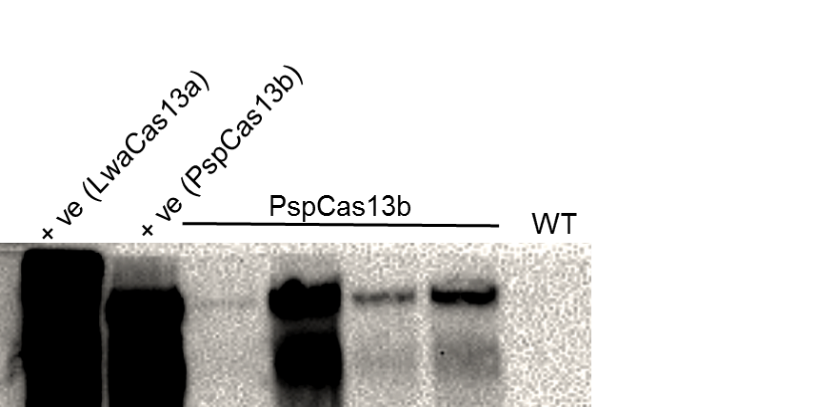

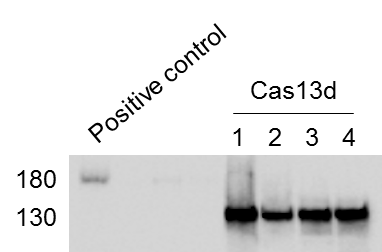


CasRx

CasRx

**Figure S4: Confirmation of Cas13 protein expression in permanent *N. benthamiana* lines.**

Total proteins were extracted from *N. benthamiana* leaves overexpressing

1. LwaCas13a-NLS , LwaCas13a-NES
2. PspCas13b
3. and CasRx-NLS

and resolved on a SDS page. Western blot was performed with anti-HA antibody. A transiently expressed LwaCas13a-NLS protein was used as positive control.

B

A

PVX-GFP

TRBO-BFP

PVX-GFP and TRBO-BFP

targeted with NS crRNA

Rep-T3

+

GFP-T2


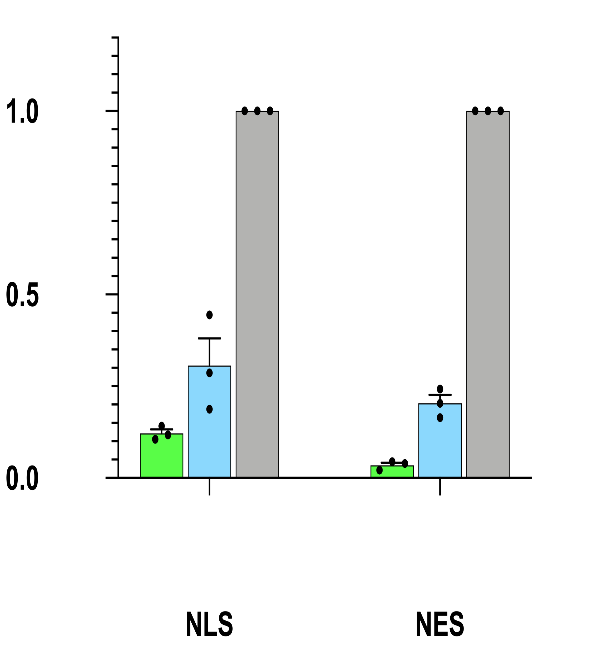


crRNA:

NS

1


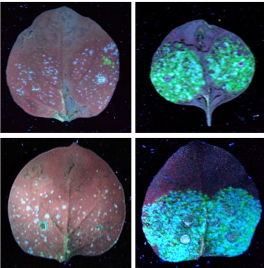


CasRx-NLS

Normalized TRBO-Rep

and PVX-GFP expression

0.5

CasRx-NES

0

TRBO-BFP + PVX-GFP

CasRx-NLS

CasRx-NES

**Figure S5: Multiplexed targeting of TRBO-BFP and PVX-GFP viruses through delivery of two different crRNAs with CasRx.**

1. GFP and BFP monitoring to assess the Cas13-mediated virus interference activities in *Agro*-infiltrated wild type *N. benthamiana* leaves in transient assays. Images were taken 3 days post infiltration. NS: non-specific crRNA; Rep: Replicase.
2. RT-qPCR analysis of TRBO-BFP and PVX-GFP simultaneous knockdown with two different crRNAs used against each virus. The transcript levels of TRBO-BFP and PVX-GFP are shown relative to the transcript level of viruses targeted with (NS) crRNA, where the transcript levels of the targeted TRBO-BFP was calculated relative to TRBO-BFP targeted with NS, and the targeted PVX-GFP was calculated relative to PVX-GFP targeted with NS. The NS value representing the values of both non-targeted viruses. Values shown as mean ± s.e.m. (n=3).

**Sequence S1:** BzCas13b amino acid sequence (3xHA-BzCas13b-NLS)

(HA and sequences are highlighted in amino acid sequences)

MYPYDVPDYAYPYDVPDYAYPYDVPDYAENKTSLGNNIYYNPFKPQDKSYFAGYFNAAMENTDSVFRELGKRLKGKEYTSENFFDAIFKENISLVEYERYVKLLSDYFPMARLLDKKEVPIKERKENFKKNFKGIIKAVRDLRNFYTHKEHGEVEITDEIFGVLDEMLKSTVLTVKKKKVKTDKTKEILKKSIEKQLDILCQKKLEYLRDTARKIEEKRRNQRERGEKELVAPFKYSDKRDDLIAAIYNDAFDVYIDKKKDSLKESSKAKYNTKSDPQQEEGDLKIPISKNGVVFLLSLFLTKQEIHAFKSKIAGFKATVIDEATVSEATVSHGKNSICFMATHEIFSHLAYKKLKRKVRTAEINYGEAENAEQLSVYAKETLMMQMLDELSKVPDVVYQNLSEDVQKTFIEDWNEYLKENNGDVGTMEEEQVIHPVIRKRYEDKFNYFAIRFLDEFAQFPTLRFQVHLGNYLHDSRPKENLISDRRIKEKITVFGRLSELEHKKALFIKNTETNEDREHYWEIFPNPNYDFPKENISVNDKDFPIAGSILDREKQPVAGKIGIKVKLLNQQYVSEVDKAVKAHQLKQRKASKPSIQNIIEEIVPINESNPKEAIVFGGQPTAYLSMNDIHSILYEFFDKWEKKKEKLEKKGEKELRKEIGKELEKKIVGKIQAQIQQIIDKDTNAKILKPYQDGNSTAIDKEKLIKDLKQEQNILQKLKDEQTVREKEYNDFIAYQDKNREINKVRDRNHKQYLKDNLKRKYPEAPARKEVLYYREKGKVAVWLANDIKRFMPTDFKNEWKGEQHSLLQKSLAYYEQCKEELKNLLPEKVFQHLPFKLGGYFQQKYLYQFYTCYLDKRLEYISGLVQQAENFKSENKVFKKVENECFKFLKKQNYTHKELDARVQSILGYPIFLERGFMDEKPTIIKGKTFKGNEALFADWFRYYKEYQNFQTFYDTENYPLVELEKKQADRKRKTKIYQQKKNDVFTLLMAKHIFKSVFKQDSIDQFSLEDLYQSREERLGNQERARQTGERNTNYIWNKTVDLKLCDGKITVENVKLKNVGDFIKYEYDQRVQAFLKYEENIEWQAFLIKESKEEENYPYVVEREIEQYEKVRREELLKEVHLIEEYILEKVKDKEILKKGDNQNFKYYILNGLLKQLKNEDVESYKVFNLNTEPEDVNINQLKQEATDLEQKAFVLTYIRNKFAHNQLPKKEFWDYCQEKYGKIEKEKTYAEYFAEVFKKEKEALIKKRPAATKKAGQAKKKK*

**Sequence S2:** BzCas13b full-length plant codon optimized DNA sequence (3x-HA-BzCas13b-NLS)

ATGTACCCATACGATGTTCCAGATTACGCTTACCCATACGATGTTCCAGATTACGCTTACCCATACGATGTTCCAGATTACGCTGAGAATAAAACTAGTTTAGGTAATAATATTTACTATAATCCTTTCAAGCCACAGGACAAATCTTATTTCGCTGGATATTTTAACGCTGCGATGGAGAATACTGACAGTGTATTTAGAGAACTTGGAAAGCGGCTTAAGGGGAAGGAGTATACATCAGAAAACTTCTTTGATGCAATTTTTAAAGAGAATATTTCGTTGGTAGAATATGAAAGATACGTCAAATTGCTATCTGATTATTTCCCTATGGCAAGACTTCTTGATAAGAAGGAAGTTCCTATAAAAGAACGAAAAGAAAACTTTAAAAAAAATTTCAAAGGTATTATTAAGGCTGTGAGAGATCTTCGAAATTTTTACACTCACAAAGAACACGGTGAAGTTGAGATCACGGATGAGATCTTTGGGGTCTTAGATGAAATGTTGAAGTCTACCGTACTAACTGTTAAGAAAAAAAAGGTCAAAACAGATAAAACCAAGGAAATTCTCAAGAAGTCCATTGAGAAGCAACTTGACATTCTTTGCCAAAAGAAACTGGAATACTTAAGGGATACAGCTAGGAAAATAGAAGAGAAAAGAAGAAATCAACGTGAGAGAGGAGAGAAGGAACTCGTGGCACCTTTTAAGTATTCAGATAAGAGGGATGATTTGATCGCTGCAATATATAACGACGCATTTGATGTTTATATAGACAAAAAAAAAGACAGTCTTAAGGAGTCTTCAAAGGCTAAGTATAATACAAAGAGTGATCCTCAACAAGAAGAAGGCGATCTTAAAATTCCTATTTCCAAGAATGGTGTTGTTTTCCTTTTGAGTCTGTTTTTGACTAAACAAGAAATACATGCTTTCAAATCAAAAATTGCTGGTTTCAAAGCAACGGTAATCGACGAAGCTACTGTTTCTGAAGCAACTGTTAGCCACGGTAAAAATAGCATATGTTTCATGGCGACGCACGAAATTTTCTCTCATCTGGCGTACAAAAAGCTCAAGCGTAAGGTAAGGACCGCAGAAATCAACTACGGAGAGGCAGAAAATGCTGAACAGCTTAGTGTTTATGCAAAGGAAACCCTGATGATGCAAATGCTTGACGAGTTGTCAAAAGTACCGGATGTGGTTTACCAAAATCTGAGTGAGGACGTGCAAAAGACTTTTATTGAAGATTGGAATGAATATCTTAAGGAAAATAACGGTGACGTAGGTACTATGGAAGAAGAGCAGGTCATTCATCCTGTCATTCGTAAGAGATATGAGGACAAATTTAATTACTTCGCGATAAGATTTCTCGATGAATTTGCGCAGTTCCCCACATTGCGATTTCAGGTTCACCTAGGCAACTACTTGCACGATTCCCGACCTAAAGAAAACCTGATCAGTGATCGGAGAATAAAGGAGAAGATTACCGTTTTTGGACGGCTTAGTGAACTGGAACACAAAAAAGCTCTTTTCATAAAAAATACAGAGACTAATGAAGATAGAGAGCATTACTGGGAAATATTCCCTAATCCGAACTACGATTTCCCCAAAGAAAATATCTCTGTAAACGATAAGGACTTTCCTATCGCAGGTTCAATTCTGGATAGGGAAAAACAACCTGTTGCTGGTAAGATTGGTATCAAGGTTAAGTTGCTTAATCAGCAATATGTAAGCGAAGTTGATAAAGCCGTTAAGGCACACCAACTAAAACAACGAAAGGCCTCTAAACCTTCGATCCAGAATATTATTGAAGAGATTGTGCCAATTAATGAATCAAATCCTAAGGAGGCAATTGTTTTTGGTGGTCAGCCGACCGCATACCTGTCTATGAATGACATACATTCTATCCTTTATGAATTTTTTGACAAATGGGAAAAAAAAAAAGAGAAACTGGAAAAAAAAGGTGAAAAGGAGCTGAGGAAAGAGATTGGTAAGGAGTTGGAAAAGAAAATTGTGGGCAAGATACAAGCTCAAATACAGCAGATAATCGACAAAGATACCAATGCAAAGATACTTAAGCCCTACCAAGATGGTAATTCAACCGCTATTGATAAGGAGAAGCTCATAAAAGACCTGAAGCAAGAACAGAATATTCTCCAGAAACTTAAGGACGAACAGACCGTGCGAGAAAAGGAGTATAACGATTTCATAGCGTACCAAGATAAGAATAGAGAGATAAACAAAGTGAGGGATAGGAATCATAAACAGTACTTAAAGGATAATTTAAAAAGAAAGTATCCCGAAGCTCCTGCACGAAAGGAAGTCCTCTATTACAGAGAGAAGGGTAAAGTTGCTGTATGGTTGGCTAACGACATTAAGCGATTTATGCCAACTGATTTTAAAAACGAATGGAAGGGCGAACAACATTCGTTGCTTCAAAAGAGCTTAGCTTATTACGAACAGTGTAAAGAAGAGCTCAAGAATTTATTACCGGAAAAAGTTTTTCAACATCTTCCTTTTAAACTTGGCGGCTACTTTCAGCAAAAGTACTTGTATCAGTTTTACACTTGTTACTTGGATAAGCGATTGGAATATATATCAGGGCTTGTTCAGCAGGCCGAAAATTTCAAGAGCGAGAACAAAGTATTCAAGAAGGTAGAGAACGAGTGCTTTAAGTTTCTTAAAAAGCAAAACTACACCCATAAAGAGCTAGATGCTAGAGTTCAGAGTATATTGGGATACCCTATTTTTCTTGAGCGTGGTTTTATGGATGAAAAACCGACCATTATAAAGGGCAAGACATTTAAGGGGAACGAAGCTCTGTTTGCAGATTGGTTCAGGTATTATAAGGAATATCAAAACTTTCAAACGTTTTACGACACTGAAAACTATCCTCTCGTAGAACTGGAAAAAAAGCAAGCAGACAGGAAGCGAAAGACCAAGATTTACCAACAGAAGAAGAACGATGTTTTTACACTGTTAATGGCTAAACATATTTTTAAGTCAGTTTTTAAGCAAGATTCTATCGATCAATTTTCTTTGGAGGATTTATACCAAAGCCGAGAAGAACGACTTGGTAATCAGGAAAGAGCAAGGCAAACTGGCGAAAGGAATACAAATTATATCTGGAACAAGACTGTTGATCTGAAACTTTGTGATGGCAAAATAACTGTCGAAAACGTGAAACTTAAAAATGTAGGAGATTTTATTAAATACGAATACGATCAAAGAGTTCAAGCTTTTCTTAAGTATGAGGAGAATATCGAATGGCAAGCCTTTTTGATCAAGGAGAGCAAAGAGGAAGAAAACTATCCTTATGTTGTGGAACGTGAGATAGAACAGTATGAGAAAGTTAGACGTGAGGAGCTTCTCAAAGAGGTCCACCTTATCGAGGAATATATTCTTGAAAAGGTAAAGGATAAGGAAATACTTAAAAAAGGCGACAACCAAAATTTTAAGTATTATATTCTCAACGGTTTGTTAAAACAGCTCAAAAATGAAGATGTAGAAAGCTATAAGGTATTTAATTTGAATACCGAACCGGAAGACGTAAATATAAACCAACTTAAGCAAGAAGCAACCGATCTAGAACAGAAAGCGTTTGTTCTCACATACATTAGGAATAAATTCGCTCATAATCAACTTCCTAAAAAGGAGTTTTGGGATTACTGTCAAGAGAAGTATGGTAAAATAGAGAAAGAGAAGACTTATGCGGAATACTTTGCCGAAGTTTTCAAAAAAGAAAAAGAGGCACTAATAAAAAAAAGGCCGGCGGCCACGAAAAAGGCCGGCCAGGCAAAAAAGAAAAAGTGA

**Map S1**: BzCas13b in *pK2GW7* (HA-BzCas13b-NLS)


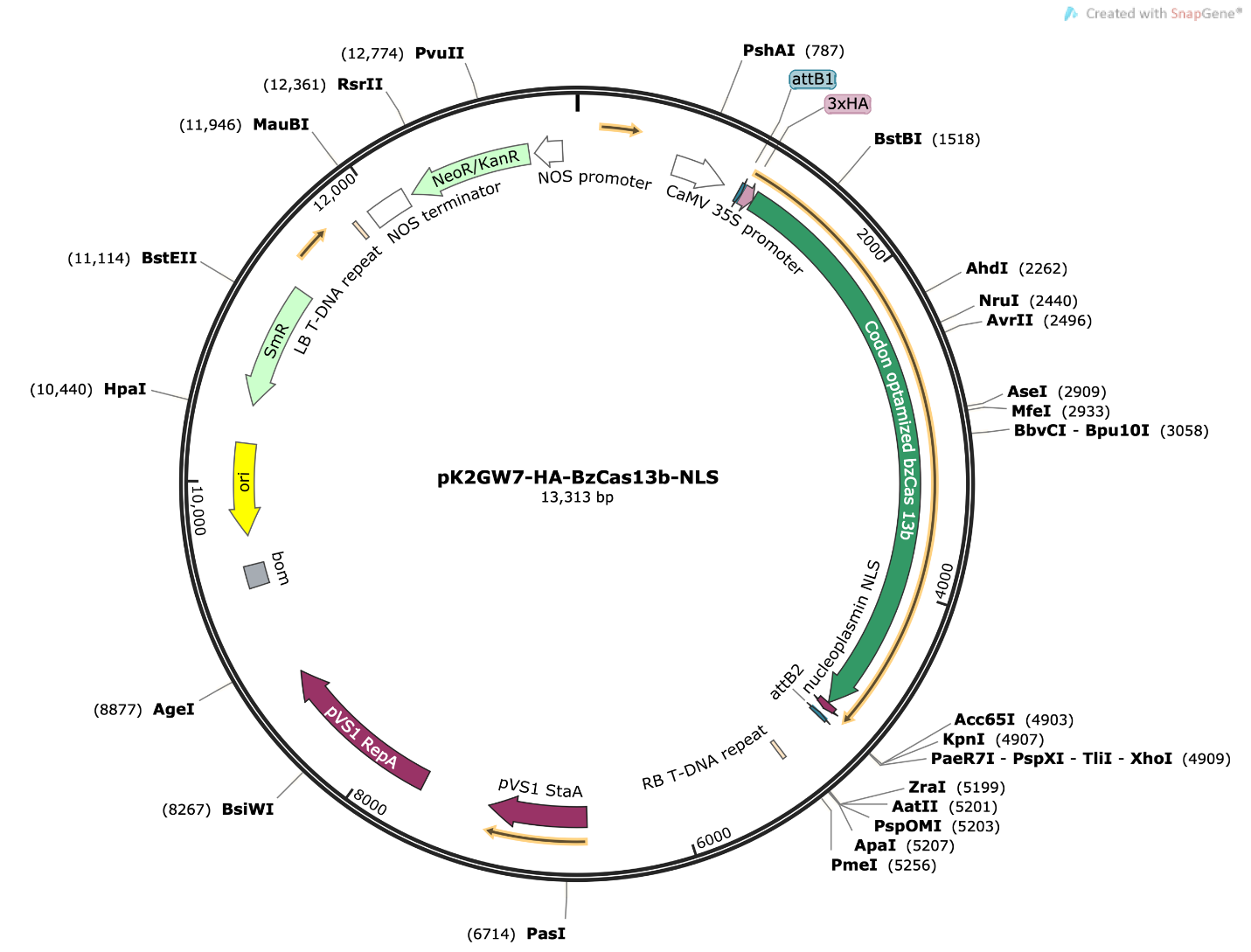


**Table S1: crRNA sequences used in this study.**

| **Name** | **Sequence (5’-3’)** | **Purpose** |
| --- | --- | --- |
| LshCas13a-TRBO-GFP-T1-F | CTAGACCACCCCAATATCGAAGGGGACTAAAACaacaggtagttttccagtagtgcaaataTTTTTTTTTg | Targeting TRBO GFP sequence |
| LshCas13a -TRBO -GFP-T1-R | gatccAAAAAAAAAtatttgcactactggaaaactacctgttGTTTTAGTCCCCTTCGATATTGGGGTGGT |  |
| LshCas13a -TRBO-GFP-T2-F | CTAGACCACCCCAATATCGAAGGGGACTAAAACtttgtctgccgtgatgtatacattgtgtTTTTTTTTTg | Targeting TRBO GFP sequence |
| LshCas13a -TRBO-GFP-T2-R | gatccAAAAAAAAAacacaatgtatacatcacggcagacaaaGTTTTAGTCCCCTTCGATATTGGGGTGGT |  |
| LshCas13a -TRBO-GFP-NS-F | CTAGACCACCCCAATATCGAAGGGGACTAAAACTTCGTCGGTAATCACCATTCCCGGCGGGTTTTTTTTTg | TRBO-GFP  Non-specific crRNA |
| LshCas13a - TRBO-GFP -NS-R | gatccAAAAAAAAACCCGCCGGGAATGGTGATTACCGACGAAGTTTTAGTCCCCTTCGATATTGGGGTGGT |  |
| LwaCas13a-TRBO-GFP-T1-TRV-F | CTAGAGATTTAGACTACCCCAAAAACGAAGGGGACTAAAACaacaggtagttttccagtagtgcaaataTTTTTTTTTg | Targeting TRBO-GFP sequence |
| LwaCas13a -TRBO-GFP-T1-R | gatccAAAAAAAAAtatttgcactactggaaaactacctgttGTTTTAGTCCCCTTCGTTTTTGGGGTAGTCTAAATCT |  |
| LwaCas13a -TRBO-GFP-T2-F | CTAGAGATTTAGACTACCCCAAAAACGAAGGGGACTAAAACtttgtctgccgtgatgtatacattgtgtTTTTTTTTTg | Targeting TRBO GFP sequence |
| LwaCas13a -TRBO-GFP-T2- R | gatccAAAAAAAAAacacaatgtatacatcacggcagacaaaGTTTTAGTCCCCTTCGTTTTTGGGGTAGTCTAAATCT |  |
| LwaCas13a-TRBO-GFP-NS -F | CTAGAGATTTAGACTACCCCAAAAACGAAGGGGACTAAAACTCCGGATCCAGAGAGATGATTCTCCCGCTTTTTTTTTg | TRBO-GFP  Non-specific crRNA |
| LwaCas13a-TRBO-GFP-NS-R | gatccAAAAAAAAAGCGGGAGAATCATCTCTCTGGATCCGGAGTTTTAGTCCCCTTCGTTTTTGGGGTAGTCTAAATCT |  |
| LwaCas13a-TRBO-Rep-T1-TRV-F | CTAGAGATTTAGACTACCCCAAAAACGAAGGGGACTAAAACcatcatcagatattccagttctaaagatTTTTTTTTTg | targeting Replicase sequence of TRBO virus |
| LwaCas13a-TRBO-Rep-T1-TRV-R | gatccAAAAAAAAAatctttagaactggaatatctgatgatgGTTTTAGTCCCCTTCGTTTTTGGGGTAGTCTAAATCT | targeting Replicase sequence of TRBO virus |
| LwaCas13a-TRBO-Rep-T2-F | CTAGAGATTTAGACTACCCCAAAAACGAAGGGGACTAAAACaaggtccagttttgaggggctattacaaTTTTTTTTTg | targeting Replicase sequence of TRBO virus |
| LwaCas13a-TRBO-Rep-T2-R | gatccAAAAAAAAAttgtaatagcccctcaaaactggaccttGTTTTAGTCCCCTTCGTTTTTGGGGTAGTCTAAATCT |  |
| LwaCas13a-TRBO-TRV-Rep-T4-F | CTAGAGATTTAGACTACCCCAAAAACGAAGGGGACTAAAACtagtacttgagcgaacaggtgtgccttgTTTTTTTTTg | targeting Replicase sequence of TRBO virus |
| LwaCas13a-TRBO-TRV-Rep-T4-R | gatccAAAAAAAAAcaaggcacacctgttcgctcaagtactaGTTTTAGTCCCCTTCGTTTTTGGGGTAGTCTAAATCT |  |
| LwaCas13a-TRBO-Rep-NS -F | CTAGAGATTTAGACTACCCCAAAAACGAAGGGGACTAAAACTCCGGATCCAGAGAGATGATTCTCCCGCTTTTTTTTTg | TRBO-GFP  Non-specific crRNA |
| LwaCas13a-TRBO-Rep-NS-R | gatccAAAAAAAAAGCGGGAGAATCATCTCTCTGGATCCGGAGTTTTAGTCCCCTTCGTTTTTGGGGTAGTCTAAATCT |  |
| LwaCas13a -TuMV-GFP-T1-F | CTAGAGATTTAGACTACCCCAAAAACGAAGGGGACTAAAACgttttccgtatgttgcatcaccttcaccct TTTTTTTTTg | Targeting TuMV-GFP sequence |
| LwaCas13a -TuMV-GFP-T1-R | gatccAAAAAAAAAagggtgaaggtgatgcaacatacggaaaacGTTTTAGTCCCCTTCGTTTTTGGGGTAGTCTAAATCT |  |
| LwaCas13a -TuMV-GFP-T2 -F | CTAGAGATTTAGACTACCCCAAAAACGAAGGGGACTAAAACcgttgggatctttcgaaagggcagattgTTTTTTTTTg | Targeting TuMV-GFP sequence |
| LwaCas13a -TuMV-GFP-T2-R | gatccAAAAAAAAAcaatctgccctttcgaaagatcccaacgGTTTTAGTCCCCTTCGTTTTTGGGGTAGTCTAAATCT |  |
| LwaCas13a-TuMV-HC pro-F | CTAGAGATTTAGACTACCCCAAAAACGAAGGGGACTAAAACccgcttgcttgtccttgggatagctcacTTTTTTTTTTg | Targeting TuMV-HC Pro sequence |
| LwaCas13a-TuMV-HC pro-R | gatccAAAAAAAAAAgtgagctatcccaaggacaagcaagcggGTTTTAGTCCCCTTCGTTTTTGGGGTAGTCTAAATCC |  |
| LwaCas13a -TuMV-CP -F | CTAGAGATTTAGACTACCCCAAAAACGAAGGGGACTAAAACtacactgaaagttccagaggttccagcgTTTTTTTTTg | Targeting TuMV-CP sequence |
| LwaCas13a -TuMV-CP-R | gatccAAAAAAAAAcgctggaacctctggaactttcagtgtaGTTTTAGTCCCCTTCGTTTTTGGGGTAGTCTAAATCT | Targeting TuMV-CP sequence |
| LwaCas13a-TuMV-NS -F | CTAGAGATTTAGACTACCCCAAAAACGAAGGGGACTAAAACTCCGGATCCAGAGAGATGATTCTCCCGCTTTTTTTTTg | TuMV-GFP  Non-specific crRNA |
| LwaCas13a-TuMV-NS-R | gatccAAAAAAAAAGCGGGAGAATCATCTCTCTGGATCCGGAGTTTTAGTCCCCTTCGTTTTTGGGGTAGTCTAAATCT |  |

| BzCas13b-TRBO-GFP-T1-F | CTAggaacaggtagttttccagtagtgcaaataGTTGGAACTGCTCTCATTTTGGAGGGTAATCACAACTTTTTTT | TRBO-GFP targeting of GFP sequence |
| --- | --- | --- |
| BzCas13b-TRBO-GFP-T1-R | tcgAAAAAAAAGTTGTGATTACCCTCCAAAATGAGAGCAGTTCCAACtatttgcactactggaaaactacctgttc |  |
| BzCas13b-TRBO-GFP-T2-F | CTAgtttgtctgccgtgatgtatacAttgtgTgGTTGGAACTGCTCTCATTTTGGAGGGTAATCACAACTTTTTTT | TRBO-GFP targeting of GFP sequence |
| BzCas13b-TRBO-GFP-T2-R | tcgAAAAAAAAGTTGTGATTACCCTCCAAAATGAGAGCAGTTCCAACcAcacaaTgtatacatcacggcagacaaa |  |
| BzCas13b-TRBO-NS-F | CTAGgtccgcttgcttgtccttgggatagctcacGTTGGAACTGCTCTCATTTTGGAGGGTAATCACAACTTTTTTTT | TRBO-GFP  Non-specific crRNA |
| BzCas13b-TRBO-NS-R | TCGAAAAAAAAAGTTGTGATTACCCTCCAAAATGAGAGCAGTTCCAACgtgagctatcccaaggacaagcaagcggac |  |
| PspCas13b-TRBO-GFP-T1-F | CTAggaacaggtagttttccagtagtgcaaatagttgtggaaggtccagttttgaggggctattacaacTTTTTTTT | TRBO-GFP targeting of GFP sequence |
| PspCas13b-TRBO-GFP-T1-R | TCGAAAAAAAAAgttgtaatagcccctcaaaactggaccttccacaactatttgcactactggaaaactacctgttc |  |
| PspCas13b-TRBO-GFP-T2-F | CTAgtttgtctgccgtgatgtatacAttgtgTggttgtggaaggtccagttttgaggggctattacaacTTTTTT | TRBO-GFP targeting of GFP sequence |
| PspCas13b-TRBO-GFP-T2-R | TCGAAAAAAAgttgtaatagcccctcaaaactggaccttccacaaccAcacaaTgtatacatcacggcagacaaa |  |
| PspCas13b-TRBO-NS-F | CTAGgtccgcttgcttgtccttgggatagctcacgttgtggaaggtccagttttgaggggctattacaacTTTTTTTT | TRBO-GFP  Non-specific crRNA |
| PspCas13b-TRBO-NS-R | TCGAAAAAAAAAgttgtaatagcccctcaaaactggaccttccacaacgtgagctatcccaaggacaagcaagcggac |  |
| CasRx-TRBO-GFP-T1-F | ctagaacccctaccaactggtcggggtttgaaacggaacaggtagttttccagtagtgcaaataTTTTTTTTT | TRBO-GFP targeting of GFP sequence |
| CasRx-TRBO-GFP-T1-R | tcgAAAAAAAAAAtatttgcactactggaaaactacctgttccgtttcaaaccccgaccagttggtaggggtt |  |
| CasRx-TRBO-GFP-T2-F | ctagaacccctaccaactggtcggggtttgaaacgtttgtctgccgtgatgtatacAttgtgTgTTTTTTTTT | TRBO-GFP targeting of GFP sequence |
| CasRx-TRBO-GFP-T2-R | tcgAAAAAAAAAAcAcacaaTgtatacatcacggcagacaaacgtttcaaaccccgaccagttggtaggggtt |  |
| CasRx-TRBO-NS-F | ctagaacccctaccaactggtcggggtttgaaacgtccgcttgcttgtccttgggatagctcacTTTTTTTT | TRBO-GFP Non-specific crRNA |
| CasRx-TRBO-NS-R | TCGAAAAAAAAAgtgagctatcccaaggacaagcaagcggacgtttcaaaccccgaccagttggtaggggtt |  |
| PspCas13b-TRBO-Rep.-T1-F | CTAGcatcatcagatattccagttctaaagatcggttgtggaaggtccagttttgaggggctattacaacTTTTTTTT | TRBO-GFP targeting of Rep sequence |
| PspCas13b-TRBO-Rep.-T1-R | TCGAAAAAAAAAgttgtaatagcccctcaaaactggaccttccacaaccgatctttagaactggaatatctgatgatg |  |
| PspCas13b-TRBO-Rep.-T2-F | CTAGgtaatttggaattctggatacgcccgggtagttgtggaaggtccagttttgaggggctattacaacTTTTTTTTTT | TRBO-GFP targeting of Rep sequence |
| PspCas13b-TRBO-Rep.-T2-R | TCGAAAAAAAAAAAgttgtaatagcccctcaaaactggaccttccacaactacccgggcgtatccagaattccaaattac |  |
| PspCas13b-RNAx TRBO-Rep-T4-F | CTAGgtagtacttgagcgaacaggtgtgccttgagttgtggaaggtccagttttgaggggctattacaacTTTTTTTTTT | TRBO-GFP targeting of Rep sequence |
| PspCas13b-RNAx TRBO-Rep-T4-R | TCGAAAAAAAAAAAgttgtaatagcccctcaaaactggaccttccacaactcaaggcacacctgttcgctcaagtactac |  |
| PspCas13b-TRBO-NS-F | CTAGgtccgcttgcttgtccttgggatagctcacgttgtggaaggtccagttttgaggggctattacaacTTTTTTTT | TRBO-GFP  Non-specific crRNA |
| PspCas13b-TRBO-NS-R | TCGAAAAAAAAAgttgtaatagcccctcaaaactggaccttccacaacgtgagctatcccaaggacaagcaagcggac |  |
| CasRx-TRBO-Rep.-T1-F | ctagaacccctaccaactggtcggggtttgaaaccatcatcagatattccagttctaaagatcgTTTTTTTTT | targeting Replicase sequence of TRBO virus |
| CasRx-TRBO-Rep.-T1-R | tcgAAAAAAAAAAcgatctttagaactggaatatctgatgatggtttcaaaccccgaccagttggtaggggtt |  |
| CasRx-TRBO-Rep.-T2-F | CTAGGaacccctaccaactggtcggggtttgaaacgtaatttggaattctggatacgcccgggtaTTTTTTTTTT | targeting Replicase sequence of TRBO virus |
| CasRx-TRBO-Rep.-T2-R | TCGAAAAAAAAAAAtacccgggcgtatccagaattccaaattacgtttcaaaccccgaccagttggtaggggttC |  |
| CasRx-RNAx TRBO-Rep-T4-F | CTAGGaacccctaccaactggtcggggtttgaaacgtagtacttgagcgaacaggtgtgccttgaTTTTTTTTTT | targeting Replicase sequence of TRBO-GFP/BFP virus |
| CasRx-RNAx TRBO-Rep-T4-R | TCGAAAAAAAAAAAtcaaggcacacctgttcgctcaagtactacgtttcaaaccccgaccagttggtaggggttC |  |
| CasRx-TRBO-NS-F | ctagaacccctaccaactggtcggggtttgaaacgtccgcttgcttgtccttgggatagctcacTTTTTTTT | TRBO-GFP  Non-specific crRNA |
| CasRx-TRBO-NS-R | TCGAAAAAAAAAgtgagctatcccaaggacaagcaagcggacgtttcaaaccccgaccagttggtaggggtt |  |
| PspCas13b-TuMV-GFP-T1-F | CTAgttttccgtatgttgcatcaccttcaccctgttgtggaaggtccagttttgaggggctattacaacTTTTTTT | TuMV-GFP targeting of GFP sequence |
| PspCas13b-TuMV-GFP-T1-R | TCGAAAAAAAAgttgtaatagcccctcaaaactggaccttccacaacagggtgaaggtgatgcaacatacggaaaa |  |
| PspCas13b-TuMV-GFP-T2-F | CTAGttcgttgggatctttcgaaagggcagattggttgtggaaggtccagttttgaggggctattacaacTTTTTTTT | TuMV-GFP targeting of GFP sequence |
| PspCas13b-TuMV-GFP-T2-R | TCGAAAAAAAAAgttgtaatagcccctcaaaactggaccttccacaaccaatctgccctttcgaaagatcccaacgaa |  |
| PspCas13b-TuMV-HC pro-F | CTAGgtccgcttgcttgtccttgggatagctcacgttgtggaaggtccagttttgaggggctattacaacTTTTTTTT | TuMV-GFP targeting of HC-Pro sequence |
| PspCas13b-TuMV-HC- pro-R | TCGAAAAAAAAAgttgtaatagcccctcaaaactggaccttccacaacgtgagctatcccaaggacaagcaagcggac |  |
| PspCas13b-TuMV-CP-F | CTAGgtacactgaaagttccagaggttccagcgtgttgtggaaggtccagttttgaggggctattacaacTTTTTTTT | TuMV-GFP targeting of CP-Pro sequence |
| PspCas13b-TuMV-CP-R | TCGAAAAAAAAAgttgtaatagcccctcaaaactggaccttccacaacacgctggaacctctggaactttcagtgtac |  |
| PspCas13b-TuMV-NS-F | CTAGgtagtacttgagcgaacaggtgtgccttgagttgtggaaggtccagttttgaggggctattacaacTTTTTTTTTT | TuMV-GFP Non-specific crRNA |
| PspCas13b-TuMV-NS-R | TCGAAAAAAAAAAAgttgtaatagcccctcaaaactggaccttccacaactcaaggcacacctgttcgctcaagtactac |  |
| CasRx-TuMV-GFP-T1-F | ctagaacccctaccaactggtcggggtttgaaacgttttccgtatgttgcatcaccttcaccctTTTTTTTT | TuMV-GFP targeting of GFP sequence |
| CasRx-TuMV-GFP-T1-R | TCGAAAAAAAAAagggtgaaggtgatgcaacatacggaaaacgtttcaaaccccgaccagttggtaggggtt |  |
| CasRx-TuMV-GFP-T2-F | ctagaacccctaccaactggtcggggtttgaaacttcgttgggatctttcgaaagggcagattgTTTTTTTT | TuMV-GFP targeting of GFP sequence |
| CasRx-TuMV-GFP-T2-R | TCGAAAAAAAAAcaatctgccctttcgaaagatcccaacgaagtttcaaaccccgaccagttggtaggggtt |  |
| CasRx-TuMV-HC pro-F | ctagaacccctaccaactggtcggggtttgaaacgtccgcttgcttgtccttgggatagctcacTTTTTTTT | TuMV-GFP targeting of HC-Pro sequence |
| CasRx-TuMV-HC pro -R | TCGAAAAAAAAAgtgagctatcccaaggacaagcaagcggacgtttcaaaccccgaccagttggtaggggtt |  |
| CasRx-TuMV-CP-F | ctagaacccctaccaactggtcggggtttgaaacgtacactgaaagttccagaggttccagcgtTTTTTTTT | TuMV-GFP targeting of CP-Pro sequence |
| CasRx-TuMV-CP-R | TCGAAAAAAAAAacgctggaacctctggaactttcagtgtacgtttcaaaccccgaccagttggtaggggtt |  |
| CasRx-TuMV-NS-F | CTAGGaacccctaccaactggtcggggtttgaaacgtagtacttgagcgaacaggtgtgccttgaTTTTTTTTTT | TuMV-GFP Non-specific crRNA |
| CasRx-TuMV-NS-R | TCGAAAAAAAAAAAtcaaggcacacctgttcgctcaagtactacgtttcaaaccccgaccagttggtaggggttC |  |
| CasRx-PVX-GFP-T2-F | ctagaacccctaccaactggtcggggtttgaaacttcgttgggatctttcgaaagggcagattgTTTTTTTT | PVX-GFP targeting of GFP sequence |
| CasRx-PVX-GFP-T2-R | TCGAAAAAAAAAcaatctgccctttcgaaagatcccaacgaagtttcaaaccccgaccagttggtaggggtt |  |
| CasRx-PVX-NS-F | CTAGGaacccctaccaactggtcggggtttgaaacgtagtacttgagcgaacaggtgtgccttgaTTTTTTTTTT | PVX-GFP  Non-specific crRNA |
| CasRx-PVX-NS-R | TCGAAAAAAAAAAAtcaaggcacacctgttcgctcaagtactacgtttcaaaccccgaccagttggtaggggttC |  |

**Table S2: primers used in this study.**

| **Name of primer** | **Sequence (5’-3’)** | **Purpose** |
| --- | --- | --- |
| LwaCas13a-NLS-TOPO-F | caccatggctagccccaaaaa | F primer to amplify LwaCas13a-NLS from Addgene plasmid #91902 |
| LwaCas13a-NLS-TOPO-R | ttaggcatagtcggggacatcat | R primer to amplify LwaCas13a-NLS from Addgene plasmid #91902 |
| LwaCas13a-NES-TOPO-F | caccatgaaagtgaccaaggtcga | R primer to amplify LwaCas13a-NES from Addgene plasmid #105815 |
| LwaCas13a-NES-TOPO-R | ttaggcatagtcggggacatcat | R primer to amplify LwaCas13a-NES from Addgene plasmid #105815 |
| PspCas13b-TOPO-F | caccGTCGTGACGTACGGCCACCATGAAC | F primer to amplify PspCas13b from Addgene plasmid #103862 |
| PspCas13b-TOPO-R | CGAATTCTTAGGCATAGTCGGGGACATC | R primer to amplify PspCas13b from Addgene 103862 |
| Cas13d-cacc topo-F | caccatgagccccaagaagaag | F primer to amplify CasRx and dCasRx from Addgene 109049 and 109050 |
| Cas13d-stop topo-R | cgaattcttacttgtacagctcg | R primer to amplify CasRx and dCasRx from Addgene 109049 and 109050 |
| CasRx-BsaI-aagg-F | CTGGGTCTCGaaggtggaggccagcatcg | F primer to remove 5' NLS sequence from CasRx-NLS and add BsaI site |
| CasRx-BsaI-tccg-R | gtcggtctcatccggatccggaattgccgg | R primer to remove 3' NLS sequence from CasRx-NLS and add BsaI site |
| 5' CACC-ATG-NES-ttcc-F | CACCATGctgtatcctgagcggctgcggcggatcctgacc | top ssDNA of 5' CACC-ATG-NES sequence to be ligated to the amplified CasRx |
| 5' CACC-ATG-NES-ttcc-R | ccttggtcaggatccgccgcagccgctcaggatacagCATGGTG | Bottom ssDNA of 5' CACC-ATG-NES sequence to be ligated to the amplified CasRx to generate 5' NES-CasRx |
| cgga-NES-HA-stop-F | CGGActgtatcctgagcggctgcggcggatcctgaccgcggccgcttacccatacgatgttccagattacgcttaa | Top ssDNA of 3' NES-HA sequence with compatible BsaI overhang to be ligated to the amplified CasRx to generate 3'CasRx-NES-HA |
| cgga-NES-HA-stop-R | ttaagcgtaatctggaacatcgtatgggtaagcggccgcggtcaggatccgccgcagccgctcaggatacag | Bottom ssDNA of 3' NES-HA sequence with compatible BsaI overhang to be ligated to the amplified CasRx to generate 3'CasRx-NES-HA |
| Final ligated CasRx-for topo-F | CACCATGctgtatcctgag | To amplify the ligation product of CasRx-NES to be moved to pENTR D Topo vector |
| Final ligated CasRx-for topo-R | ttaagcgtaatctggaacatcg | To apmlify the ligation product of CasRx-NES to be moved to pENTR D Topo vector |
| BFP to TRBO-AseI-F | gtcattaattaagccaccatggtgagcaagggcg | F primer to amplify EBFP encoding sequence from LeGO-EBFP2 plasmid |
| BFP to TRBO-NotI-R | gtagcggccgcggtccctcgacgaattcttacttg | R primer to amplify EBFP encoding sequence from LeGO-EBFP2 plasmid |
| pJL-TRBO-Rep-qPCR-F | cagatgttcacactgtgcatg | F primer of qPCR for TRBO Rep sequence |
| pJL-TRBO-Rep-qPCR-R | ccttgacaatgcgaccaaa | R primer of qPCR for TRBO Rep sequence |
| TRBO-GFP qPCR-F | CCAGACAACCATTACCTGTCG | F primer of qPCR for TRBO GFP sequence |
| TRBO-GFP qPCR-R | GCTCATCCATGCCATGTGTA | R primer of qPCR for TRBO GFP sequence |
| TuMV/PVX-GFP qPCR-F | gaacttttcactggagttgtcccaat | F primer of qPCR for GFP sequence of TuMV-GFP and PVX-GFP |
| TuMV/PVX-GFP qPCR-R | ggaacaggtagttttccagtagtgc | R primer of qPCR for GFP sequence of TuMV-GFP and PVX-GFP |
