# Supplementary material for: CRISPR-Cas13d mediates robust RNA virus interference in plants
Source: Genome Biol. 2019 Dec 2;20:263. doi: 10.1186/s13059-019-1881-2 (PMC6886189; doi:10.1186/s13059-019-1881-2)
Supplement: Supplementary file 2 — Additional file 2. Review history. [file 13059_2019_1881_MOESM2_ESM.docx]

Review History

**First round of review**

**Reviewer 2**

**Are you able to assess all statistics in the manuscript, including the appropriateness of statistical tests used?**

Yes, and I have assessed the statistics in my report.

**Comments to author:**

I read the manuscript of Mahas et al with great interest. The authors characterize three different Cas13 nuclease (a, b and d) in how efficient they can be used to target viral RNA in plant cells. In previous work also published in genome biology the group of Magdy Mahfouz was already able to show that LshCas13a can be used for this purpose, although not is a very high efficiency.  Now they include in their new study further Cas13 homologues that became available only more recently. Interesting, the application of both lead to a much better suppression of viral replication. Intriguingly, this was true for proteins fused to either NLS or NES sequences. Especially with Cas13d (CasRx) shows an impressive high efficiency in blocking viral replication, also when an expression cassette is stably integrated in to the plant genome. Moreover, using a clever experimental  setup, based on two noninterfering RNA viruses, they find no indication that the enzyme shows a general unspecific collateral RNAse activity. The data are clear and convincing, all necessary controls were included. The result in of broad interest not only to plant molecular biologist as they demonstrate which enzyme is best suited for use, most probably not only to combat viral replication but also mRNA degradation. This will safe many researchers a lot of time and efforts. 
A few question remain the authors should take into account:  P 12 line 15 ff:
How many transgenic lines were produced per construct?
Did all lines behave in a similar way to induce viral resistance?
Did the transgenic plants show any growth retardation due to the transgenes?

**Authors Response**

**Point-by-point responses to the reviewers’ comments:**

Reviewer #2: 

• I read the manuscript of Mahas et al with great interest. The authors characterize three different Cas13 nuclease (a, b and d) in how efficient they can be used to target viral RNA in plant cells. In previous work also published in genome biology the group of Magdy Mahfouz was already able to show that LshCas13a can be used for this purpose, although not is a very high efficiency. Now they include in their new study further Cas13 homologues that became available only more recently. Interesting, the application of both lead to a much better suppression of viral replication. Intriguingly, this was true for proteins fused to either NLS or NES sequences. Especially with Cas13d (CasRx) shows an impressive high efficiency in blocking viral replication, also when an expression cassette is stably integrated in to the plant genome. Moreover, using a clever experimental setup, based on two noninterfering RNA viruses, they find no indication that the enzyme shows a 
general unspecific collateral RNAse activity. The data are clear and convincing, all necessary controls were included. The result in of broad interest not only to plant molecular biologist as they demonstrate which enzyme is best suited for use, most probably not only to combat viral replication but also mRNA degradation. This will safe many researchers a lot of time and efforts. 

*o We thank the reviewer for the careful examination of our manuscript and his/her interest in our work.* 

• A few questions remain the authors should take into account: P 12 line 15 ff: 

• How many transgenic lines were produced per construct? 

*o We generated between 7 to 10 transgenic plants per each Cas13 construct. Out of these plants, we confirmed Cas13 protein expression of 4 to 6 plants for each variant.* 

• Did all lines behave in a similar way to induce viral resistance? 

*o Yes, we tested at least 3 lines for each construct in our virus interference assays, and all showed relatively similar interference efficiencies compared to controls.* 

• Did the transgenic plants show any growth retardation due to the transgenes? 

*o We thank the reviewer for this critical insight. Along the way in this project, we observed minimal growth retardation and/or morphological differences in few of the transgenic LwaCas13a-NES and PspCas13b plants compared to wild type Nicotiana benthamiana plants. However, we picked the lines that had normal and similar growth to wild type plants for our RNA virus interference experiments. In other words, the Cas13 overexpression lines we used for our RNA virus interference experiments are phenotypically indistinguishable from the wild type plants.*
